# IL7Rα, but not Flk2, is required for hematopoietic stem cell reconstitution of tissue-resident lymphoid cells

**DOI:** 10.1242/dev.200139

**Published:** 2022-01-24

**Authors:** Atesh K. Worthington, Taylor Cool, Donna M. Poscablo, Adeel Hussaini, Anna E. Beaudin, E. Camilla Forsberg

**Affiliations:** 1 Institute for the Biology of Stem Cells, University of California, Santa Cruz, Santa Cruz, CA 95064, USA; 2 Program in Biomedical Science and Engineering: Department of Molecular, Cell, and Developmental Biology, University of California, Santa Cruz, Santa Cruz, CA 95064, USA; 3 Department of Biomolecular Engineering, University of California, Santa Cruz, Santa Cruz, CA 95064, USA

**Keywords:** Hematopoiesis, IL7R, Lineage tracing, Hematopoietic stem cells, Transplantation, Tissue-resident lymphocytes

## Abstract

Tissue-resident lymphoid cells (TLCs) span the spectrum of innate-to-adaptive immune function. Unlike traditional, circulating lymphocytes that are continuously generated from hematopoietic stem cells (HSCs), many TLCs are of fetal origin and poorly generated from adult HSCs. Here, we sought to further understand murine TLC development and the roles of Flk2 and IL7Rα, two cytokine receptors with known function in traditional lymphopoiesis. Using Flk2- and Il7r-Cre lineage tracing, we found that peritoneal B1a cells, splenic marginal zone B (MZB) cells, lung ILC2s and regulatory T cells (Tregs) were highly labeled. Despite high labeling, loss of Flk2 minimally affected the generation of these cells*.* In contrast, loss of IL7Rα, or combined deletion of Flk2 and IL7Rα, dramatically reduced the number of B1a cells, MZBs, ILC2s and Tregs, both *in situ* and upon transplantation, indicating an intrinsic and essential role for IL7Rα. Surprisingly, reciprocal transplants of wild-type HSCs showed that an IL7Rα^−/−^ environment selectively impaired reconstitution of TLCs when compared with TLC numbers *in situ*. Taken together, our data defined Flk2- and IL7Rα-positive TLC differentiation paths, and revealed functional roles of Flk2 and IL7Rα in TLC establishment.

## Abstract (precis)

**Summary:** Tissue-resident lymphoid cells develop via IL7Rα-positive progenitors and are repopulated by transplanted adult hematopoietic stem cells; however, such TLC lymphopoiesis cannot be fully rescued in IL7Rα^−/−^ recipient mice.

## INTRODUCTION

Traditional circulating immune cells are typically defined as either myeloid or lymphoid and generated from hematopoietic stem cells (HSCs). Myeloid cells are involved in rapid, broad response innate immunity whereas traditional lymphoid cells are involved in slow, specific adaptive immunity (Bonilla and Oettgen, 2010; Netea et al., 2011; Beaudin and Forsberg, 2016; Cool and Forsberg, 2019). The adaptive immune response relies on the complex B and T cell receptor repertoire generation. Although this dichotomy of immune cells is clear for circulating cells, it is unclear whether tissue-resident immune cells squarely belong to the myeloid or lymphoid lineage, with many recent studies alluding to complex and dynamic origins (Beaudin and Forsberg, 2016; Beaudin et al., 2016; Leung et al., 2019). Unlike circulating immune cells that traffic to non-lymphoid organs upon activation, tissue-resident immune cells reside in non-lymphoid organs, do not recirculate and have specialized functions that span the innate-adaptive spectrum (Davies et al., 2013; Chou and Li, 2018). For example, B1a cells in the peritoneal cavity are considered B cells (Kantor and Herzenberg, 1993). However, they do not undergo the same B cell receptor selection process as circulating B cells and are thus deemed an ‘innate-like’ lymphoid cell. Although recent studies have rapidly propelled the understanding of tissue macrophage specification and function (Blériot et al., 2020; Martin and Gurevich, 2021), it is unclear whether tissue-resident lymphoid cell (TLC) differentiation is orchestrated similarly to circulating lymphoid cells; here, we sought to determine whether their differentiation is regulated by classic lymphoid genes.

**Fig. 1.**
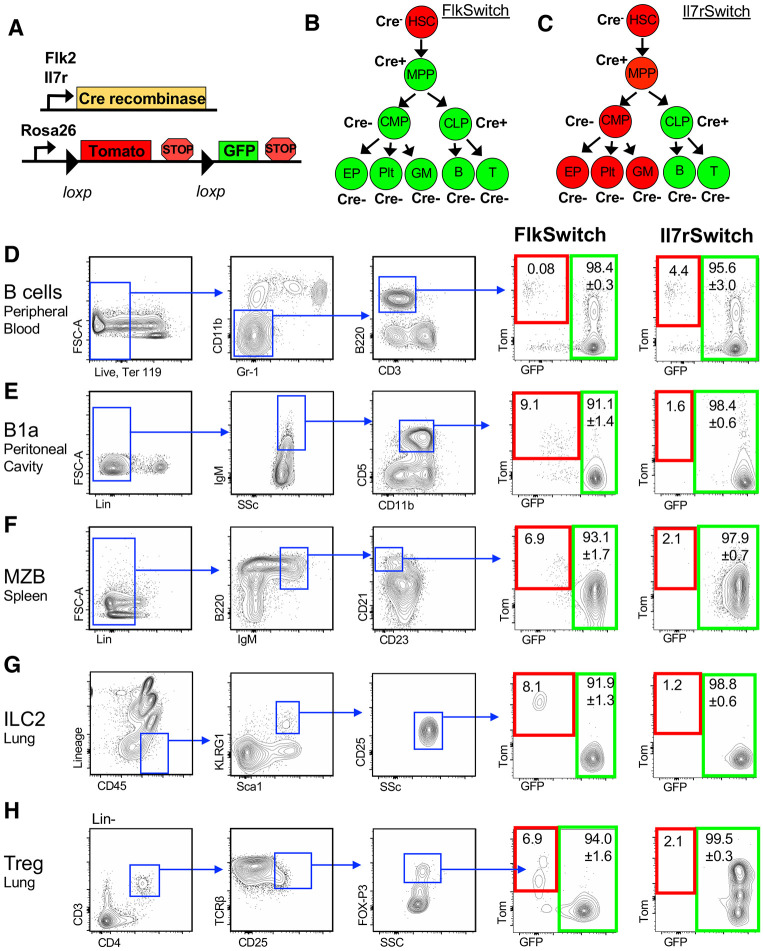
**Flk2-Cre and IL7R-Cre efficiently labeled tissue-resident lymphoid populations.** (A) Genetics of the ‘Switch’ models. Flk2 or IL7R regulatory elements drive Cre recombinase expression. Flk2-Cre or IL7r-Cre mice were crossed to Rosa26^mTmG^ mice expressing a dual color reporter expressing either Tomato (Tom) or GFP. (B,C) Schematics of Cre-mediated reporter switching in the ‘FlkSwitch’ (B) and ‘IL7rSwitch’ (C) models. Expression of Cre results in an irreversible switch from Tomato to GFP expression. Once a cell expresses GFP, it can only give rise to GFP-expressing progeny. These models represent Cre-driven labeling in young adult steady-state hematopoiesis of circulating traditional mature myeloid and lymphoid cells. (D-H) TLCs were highly labeled in both FlkSwitch and IL7rSwitch lineage tracing models. Representative flow cytometric analysis of reporter expression across traditional circulating B cells and TLCs, all pre-gated on singlets, lymphocytes and live cells. (D) Traditional B cells (Ter119^−^ CD11b^−^ Gr-1^−^ CD3^−^ B220^+^) in the peripheral blood and different tissue-resident lymphoid populations in adult mice. (E) B1a (Lin^−^ IgM^+^ CD5^+^ CD11b^mid^) cells in the peritoneal cavity. (F) MZB cells (Lin^−^ B220^+^ IgM^+^ CD21^+^ CD23^−^) in the spleen. (G) ILC2 (Lin^−^ CD45^+^ KLRG^+^ Sca-1^+^ CD25^+^) in the lung. (H) Tregs (Lin^−^ CD3^+^ CD4^+^ TCRB^+^ CD25^+^ FOX-P3^+^) in the lung. Tomato and GFP expression are highlighted by red and green boxes, respectively, in FlkSwitch and IL7RSwitch models. Values indicate mean frequencies±s.e.m. of gated Flk2-Cre and IL7R-Cre marked GFP^+^ populations. Data are representative of 4-5 mice per cohort in three independent experiments.

We were particularly interested in the cytokine receptors Flk2 (also known as Flt3) and IL7Rα because they are required for traditional adult lymphopoiesis. This finding was demonstrated by impaired lymphopoiesis in both Flk2^−/−^ and Il7rα^−/−^ mice and supported by Flk2-Cre and Il7rα-Cre driven lineage tracing of cells with increasingly restricted lymphoid potential (Peschon et al., 1994; Kikuchi et al., 2008; Beaudin et al., 2014; Leung et al., 2019) (Fig. 1A,B,C). As previously described (Boyer et al., 2011, 2012; Beaudin et al., 2016; Leung et al., 2019; Cool et al., 2020), these models were generated by crossing mice expressing Flk2-Cre (Benz et al., 2008) or Il7r-Cre (Schlenner et al., 2010) to mTmG mice expressing a dual-color fluorescent reporter (Muzumdar et al., 2007), creating the ‘FlkSwitch’ and ‘Il7rSwitch’ models (Fig. 1A). In both models, cells express Tomato (Tom) until Cre-mediated recombination results in the irreversible switch to GFP expression by that cell and all of its downstream progeny (Fig. 1B,C). We previously demonstrated that Il7r-Cre labeling is not constrained to lymphoid cells. Surprisingly, in the ‘Il7rSwitch’ lineage tracing model, fetally derived adult tissue-resident macrophages (trMacs), were highly labeled and IL7Rα was required for early trMac development (Leung et al., 2019), whereas Flk2-Cre did not efficiently label trMacs (Hoeffel et al., 2015; Hashimoto et al., 2013; Epelman et al., 2014; Leung et al., 2019). We have also previously examined lung eosinophils, another myeloid cell type; interestingly, despite the high Flk2-Cre and minimal Il7r-Cre labeling of eosinophils, they depend on cell extrinsic IL7Rα, but not Flk2 (Cool et al., 2020). The development and generation of these tissue-resident myeloid cells evidently does not follow that of other traditional circulating myeloid cells. Similarly, many innate-like lymphoid cells are thought to arise from fetal progenitors (Beaudin and Forsberg, 2016) and it remains unclear whether their development follows traditional lymphoid paths and whether they are functionally regulated by known lymphoid drivers. For example, there is evidence that many TLCs arise via common lymphoid progenitor (CLP)-independent pathways (Ghaedi et al., 2016), and there is differential requirement for Flk2 and IL7Rα amongst different hematopoietic cell types (Sitnicka et al., 2007; Beaudin et al., 2014). To determine whether Flk2 and IL7Rα are involved in TLC development, we employed lineage tracing, germline knockouts and HSC transplantation assays.

## RESULTS

### Flk2-Cre and Il7r-Cre highly label tissue-resident lymphoid cell populations

To test whether TLCs arise via differentiation pathways similar to circulating lymphocytes, we examined labeling of TLCs in the FlkSwitch and Il7rSwitch lineage tracing models (Fig. 1A-C). We compared reporter expression of traditional peripheral blood B cells with reporter expression of B1a cells isolated from the peritoneal cavity, marginal zone B cells (MZB) from the spleen, type 2 innate lymphoid cells (ILC2) from the lung and regulatory T cells (Treg) from the lung (Fig. 1D-H). As expected, and previously reported, Cre-driven labeling of circulating peripheral blood B cells was greater than 95% in both FlkSwitch and Il7rSwitch mice (Fig. 1D) (Schlenner et al., 2010; Leung et al., 2019). Similarly, Cre-driven labeling was uniformly high for all TLCs examined, a trend we also observed in B1b and B2 cells (Fig. S1A). Together, our data showed that B1a cells, ILC2, MZB and Tregs arise from Flk2- and IL7Rα-positive cells, as do traditional circulating lymphoid cells.

### Tissue-resident lymphoid cells are severely reduced in the absence of IL7Rα, but not Flk2

**Fig. 2.**
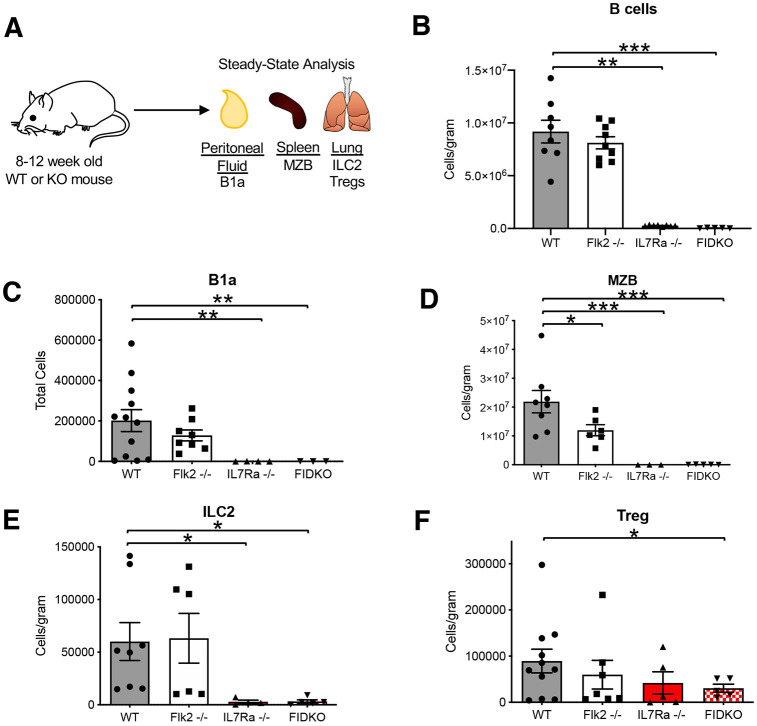
**Tissue-resident lymphoid cells are severely reduced in the absence of IL7Rα, but not Flk2.** (A) Schematic of experimental design: peritoneal fluid, spleen and lung from 8-12 week old WT, Flk2^−/−^, IL7Rα^−/−^ and FIDKO HSCs were harvested and analyzed for cellularity of TLCs and represented in the following bar plots of WT (gray), Flk2^−/−^ (white), IL7Rα^−/−^ (red) and Flk2^−/−^/IL7Rα^−/−^ double knockout (FIDKO; red/white). (B) Traditional B cells in the lung were significantly reduced in both IL7Rα^−/−^ and FIDKO mice, but not in Flk2^−/−^ mice. Quantification of B cells (Live, Ter119^−^ Mac1^−^ Gr1^−^ CD19^+^) per gram of tissue in the lungs. (C) B1a cells in the peritoneal cavity were significantly reduced in IL7Rα^−/−^ and FIDKO mice compared with WT. Quantification of total cell numbers in the peritoneal cavity. (D) MZBs were significantly reduced in Flk2^−/−^, IL7Rα^−/−^ and FIDKO compared with WT mice. Quantification of cells/gram of tissue in the spleen. (E) ILC2s were significantly reduced in IL7Rα^−/−^ and FIDKO compared with WT mice. Quantification of cells/gram of tissue in the lung. (F) Tregs were significantly reduced only in FIDKO compared with WT mice. Quantification of cells/gram of tissue in the lung. WT *n*=8 (all male), Flk2^−/−^ *n*=6 (all male), IL7Rα^−/−^ *n*=4 (all male), FIDKO *n*=5 (3 male); representing four independent experiments, mean±s.e.m. Differences were analyzed with unpaired two-tailed Student's *t*-test: *P<0.05, **P<0.005, ***P<0.0005.

We and others have previously shown that loss of Flk2 results in a reduction of hematopoietic progenitor cells, with a less severe reduction in mature B cells in the peripheral blood (Mackarehtschian et al., 1995; Sitnicka et al., 2007; Jensen et al., 2008; Beaudin et al., 2014). The loss of IL7Rα has also been shown to result in a severe reduction of peripheral blood B cells (Peschon et al., 1994). Here, we observed similar trends in the lung, in which traditional CD19^+^ B cells, although not significantly reduced in Flk2^−/−^ mice, were significantly reduced in the IL7Rα^−/−^ mice (Fig. 2A,B). Similar trends were observed in peripheral blood CD3^+^ T cells (Fig. S2A). To determine whether TLCs were similarly affected, we quantified their cellularity in the absence of Flk2 and IL7Rα. Compared with wild-type (WT) numbers of each cell type, only MZBs were significantly reduced in the absence of Flk2 (Fig. 2D), whereas B1a cells, ILC2s and MZBs were all significantly reduced in the absence of IL7Rα (Fig. 2C-E), consistent with previous studies of these cells in the same and other tissues, and peritoneal B1b and B2 cells (Fig. S1B,B′) (Hesslein et al., 2006; Ghosn et al., 2012; Patton et al., 2014; Robinette et al., 2017). Interestingly, despite being as efficiently labeled by both Flk2-Cre and IL7r-Cre (Fig. 1H), Tregs were not significantly reduced in either Flk2^−/−^ or IL7Rα^−/−^ mice, although there was a downward trend (Fig. 2F), as previously seen in thymic and splenic Tregs of IL7Rα^−/−^ mice (Bayer et al., 2008). We hypothesized that Flk2 may compensate for the lack of IL7Rα, and vice versa, allowing for near-normal Treg development in the absence of either receptor. To test this hypothesis, we generated Flk2, IL7Rα double knockout (FIDKO) mice and quantified total Tregs in the lung. We found a significant reduction of Tregs in the lung of FIDKO mice compared with WT mice (Fig. 2F), revealing an overlapping and cell-specific role of Flk2 and IL7Rα. We also quantified B1as, ILC2s and MZBs in the FIDKO mice and found that they recapitulated the severe reductions in numbers that we found in the IL7Rα^−/−^ mice. These data suggest that both Flk2 and IL7Rα do have functional roles in TLC development during steady-state hematopoiesis, although IL7Rα appears to be more important than Flk2, as indicated by the more severe reduction in cell numbers.

### Flk2^−/−^ and IL7Rα^−/−^ HSCs have impaired tissue-resident lymphoid cell reconstitution

**Fig. 3.**
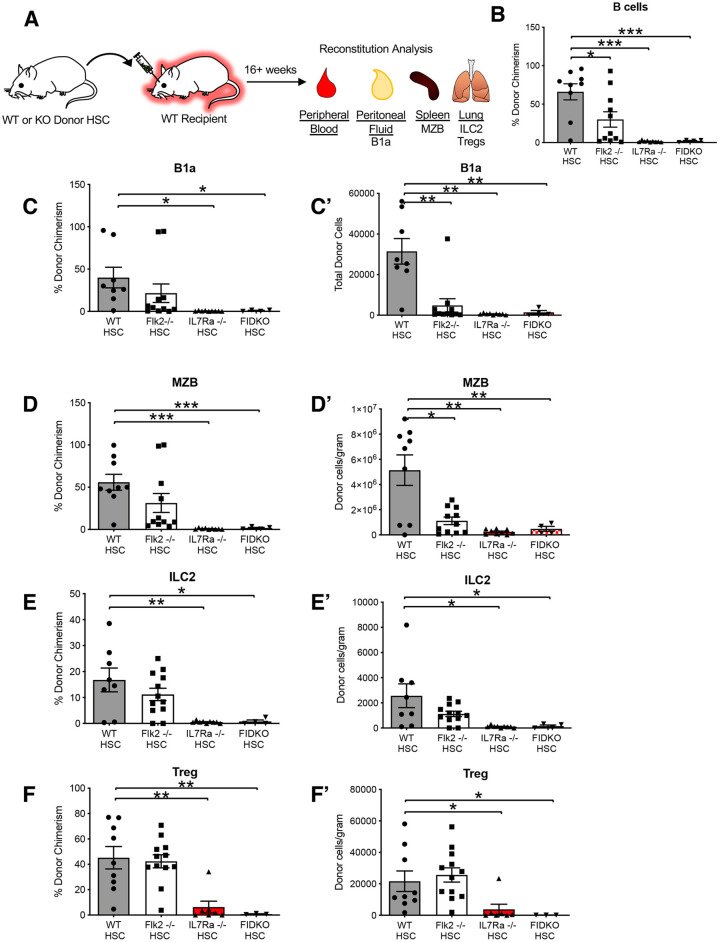
**IL7Rα^−/−^ and Flk2^−/−^ HSCs had impaired tissue-resident lymphoid cell reconstitution compared with WT HSCs.** (A) Schematic of experimental design: 500 WT, Flk2^−/−^, IL7Rα^−/−^ or FIDKO HSCs were transplanted into sublethally irradiated fluorescent WT recipients (mTmG or UBC-GFP) and donor contribution of TLCs was quantified 16 weeks post transplantation. (B) Reconstitution of peripheral blood B cells was significantly impaired upon transplantation of Flk2^−/−^ (white bars), IL7Rα^−/−^ (red bars) or FIDKO HSCs (red/white bars) compared with WT HSCs (gray bars). Quantification of donor chimerism of traditional B cells in the peripheral blood. (C-F) Donor chimerism of TLCs significantly reduced from IL7Rα^−/−^ (red bars) and FIDKO (red square bars) donor HSCs compared with WT (gray bars) donor HSCs. Quantification of donor chimerism of B1a cells (C), MZBs (D), ILC2s (E) and Tregs (F). (C′-F′) Cellularity of B1a and MZB cells were significantly reduced from Flk2^−/−^ (white bars), IL7Rα^−/−^ (red bars) and FIDKO (red/white bars) donor HSCs compared with WT (gray bars) donor HSCs. Cellularity of lung ILC2 and Tregs were significantly reduced from IL7Rα^−/−^ (red bars) and FIDKO (red/white bars), but not Flk2^−/−^ (white bars), donor HSCs compared with WT (gray bars) donor HSCs. Quantification of total B1a cells (C′), cells/gram spleen of MZBs (D′), cells/gram lung ILC2s (E′) and cells/gram of lung tissue Tregs (F′). WT *n*=8 (6 male), Flk2^−/−^ *n*=12 (8 male), IL7Rα^−/−^ *n*=7 (4 male), FIDKO *n*=4 (3 male), representing a minimum of four independent experiments, mean±s.e.m. Differences were analyzed with unpaired two-tailed Student's *t*-test: *P<0.05, **P<0.005, ***P<0.0005.

Hematopoietic stem and progenitor cell (HSPC) differentiation relies on cytokine receptors such as Flk2 and IL7R to receive instructive signals from the environment (Zhang and Lodish, 2008). For example, we have previously shown that Flk2 is intrinsically required on HSPCs for both myeloid and lymphoid reconstitution, as demonstrated by the reduced capacity of Flk2^−/−^ HSCs to generate myeloid and lymphoid progeny, including traditional circulating lymphoid cells, upon transplantation into a WT host (Beaudin et al., 2014). More recently, we demonstrated an extrinsic requirement of IL7Rα in the generation of eosinophils from WT HSCs, as demonstrated by a reduced capacity of WT HSCs to generate eosinophils upon transplantation into an IL7Rα^−/−^ host (Cool et al., 2020). Therefore, we were curious whether the deficiency in TLCs we observed in Flk2^−/−^, IL7Rα^−/−^ and FIDKO mice (Fig. 2) is due to the cell-intrinsic lack of receptors or due to changes to surrounding cells. To begin to answer this question, we transplanted WT, Flk2^−/−^, IL7Rα^−/−^ or FIDKO HSCs into sublethally irradiated WT hosts (Fig. 3A). Under such transplantation conditions, host cells are partially, but not completely, depleted (Boyer et al., 2019), leading to robust and long-term HSC engraftment (Fig. S3). We first examined donor chimerism of circulating lymphoid cells. As expected, peripheral blood B cell donor chimerism was significantly lower from Flk2^−/−^ donor HSCs, IL7Rα^−/−^ HSCs and FIDKO HSCs compared with WT HSCs (Fig. 3B); similar results were observed for peripheral blood T cells (Fig. S2B), whereas granulocyte/macrophage (GM) reconstitution was significantly impaired only from Flk2^−/−^ donor HSCs (Fig. S3A). When examining TLCs, we observed only donor chimerism of IL7Rα^−/−^ and FIDKO HSCs was significantly impaired, whereas Flk2 deficiency alone did not result in significant differences (Fig. 3C-F′; Fig. S1C,C″). A limitation of donor chimerism is that this measure relies on host cell numbers and therefore makes it more difficult to determine the intrinsic requirement of these receptors because the number of host cells changes dynamically over time post-conditioning (Boyer et al., 2019). To overcome this limitation, we quantified the absolute cellularity of donor-derived cells with a bead-based method that allows for simultaneous flow cytometric analysis and counting, which is purely a measure of donor cells (Boyer et al., 2019; Rajendiran et al., 2020; Poscablo et al., 2021). Quantification of absolute cellularity of donor-derived cells between WT and knockout HSCs revealed that reconstitution of B1a cells (Fig. 3C′) and MZBs (Fig. 3D′) was also significantly impaired by the loss of Flk2 alone. Regardless of quantification method, deletion of either IL7Rα alone, or both IL7Rα and Flk2, led to impaired reconstitution of all TLCs examined (Fig. 3C′-F′). This was intriguing as we did not observe significant reduction of Tregs in IL7Rα^−/−^ at steady state (Fig. 2F). This is likely due to the stress of transplantation, which reveals more dynamic requirement of IL7Rα – we previously observed a similar trend when we compared GM at steady state in Flk2^−/−^ mice to transplantation of Flk2^−/−^ HSCs (Beaudin et al., 2014). These data suggest that IL7Rα is required cell intrinsically for TLC reconstitution, that Flk2 cannot compensate for this requirement and that IL7Rα is not capable of fully compensating for the loss of Flk2.

### WT HSCs have enhanced tissue-resident lymphoid cell reconstitution capacity in an IL7Rα^−/−^ environment

**Fig. 4.**
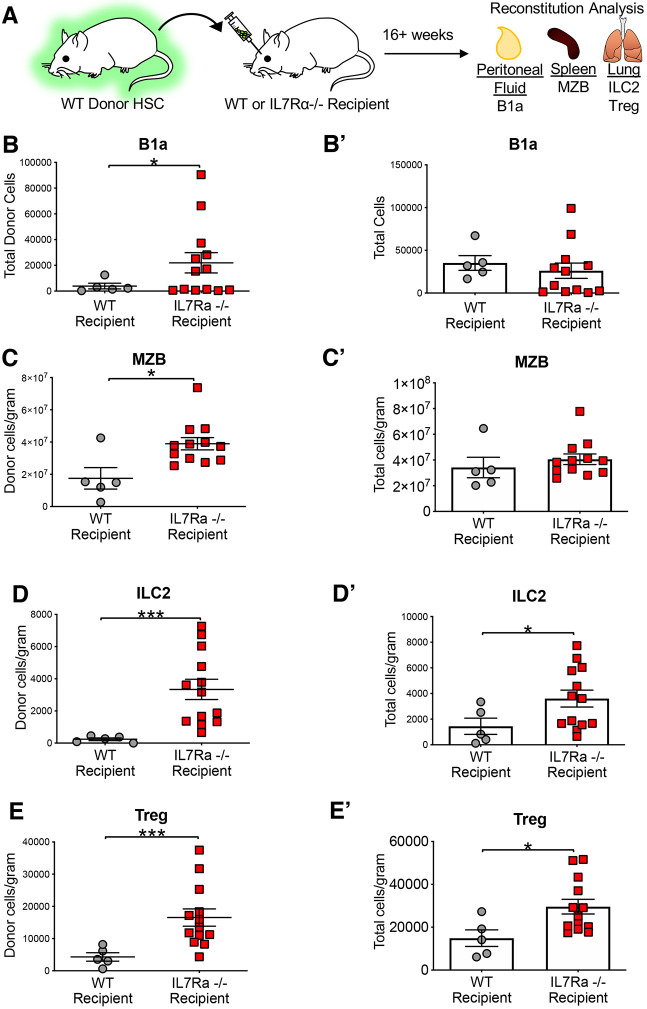
**WT HSCs have enhanced non-traditional lymphoid cell reconstitution capacity in an IL7Rα^−/−^ environment.** (A) Schematic of experimental design: 500 fluorescent WT HSCs (UBC-GFP) were transplanted into sublethally irradiated non-fluorescent WT or IL7Rα^−/−^ recipients. Donor contribution to TLCs was quantified 16 weeks post-transplantation. (B-E) Significantly greater donor-derived TLCs in an IL7Rα^−/−^ recipient (red squares) compared with a WT recipient (gray dots). Quantification of donor-derived cells of B1a cells (B), ILC2s (D), MZBs (C) and Tregs (E). (B′-E′) Significantly greater total ILC2 and Treg cells in an IL7Rα^−/−^ recipient (red squares) compared with a WT recipient (gray dots). Quantification of total cells (donor+host) of B1a cells (B′), ILC2s (D′), MZBs (C′) and Tregs (E′). WT recipient *n*=5 (1 male), IL7Rα^−/−^ recipient *n*=13 (2 male), representing four independent experiments, mean±s.e.m. Differences were analyzed with unpaired two-tailed Student's *t*-test: *P<0.05, ***P<0.0005.

Although the HSC transplantations of Fig. 3 demonstrated a cell intrinsic requirement of IL7Rα for TLC reconstitution, it is also possible that extrinsic IL7Rα may be required, as we previously demonstrated for eosinophil reconstitution (Cool et al., 2020). To test this, we performed transplantation of WT HSCs into IL7Rα-deficient mice (Fig. 4A). HSC engraftment in the bone marrow (BM) was similar between WT and IL7Rα^−/−^ recipients (Fig. S3B), consistent with the lack of IL7R expression and function in HSCs. Surprisingly, we observed a significantly greater number of donor-derived B1a cells, MZBs, ILC2s and Tregs (Fig. 4B-E′), as well as B1b and B2 cells (Fig. S1D,D″), but not circulating T (Fig. S2C) or myeloid cells (Fig. S3C), in the IL7Rα^−/−^ hosts compared with WT hosts. This raised the question of whether the greater number of donor-derived cells in the IL7Rα^−/−^ mice was reflective of an overall greater number of cells, or whether the reconstitution by WT HSCs only compensated for the lower cell numbers of cells in the IL7Rα^−/−^ host. Therefore, we determined the total number of host- and donor-derived cells and compared these numbers between WT hosts and IL7Rα^−/−^ hosts. We found no significant differences between WT and IL7Rα^−/−^ recipients of total B1a (Fig. 4B′) and MZB (Fig. 4C′) cells, B1b and B2 cells (Fig. S1D′,D‴) or peripheral blood T cells (Fig. S2C′). Interestingly, we observed significantly greater total numbers of ILC2s (Fig. 4D′) and Tregs (Fig. 4E′) in the IL7Rα^−/−^ recipients compared with the WT hosts. Designation of each individual across experiments failed to reveal evidence of sex-specific trends or differences (Fig. S4A-C′), despite a previous report of differential KLRG1 receptor expression in ILC2s in males and females (Kadel et al., 2018). Thus, the enhanced reconstitution of tissue-resident T lymphocytes in an IL7Rα^−/−^ environment compared with consistent reconstitution of tissue-resident B lymphocytes suggests differential dependence on IL7/IL7R signaling in the development of these cell types (Schluns et al., 2000; Guimond et al., 2009; Osborne et al., 2011).

### WT HSCs transplanted into IL7Rα^−/−^ mice are not fully capable of rescuing the impaired lymphoid phenotype

**Fig. 5.**
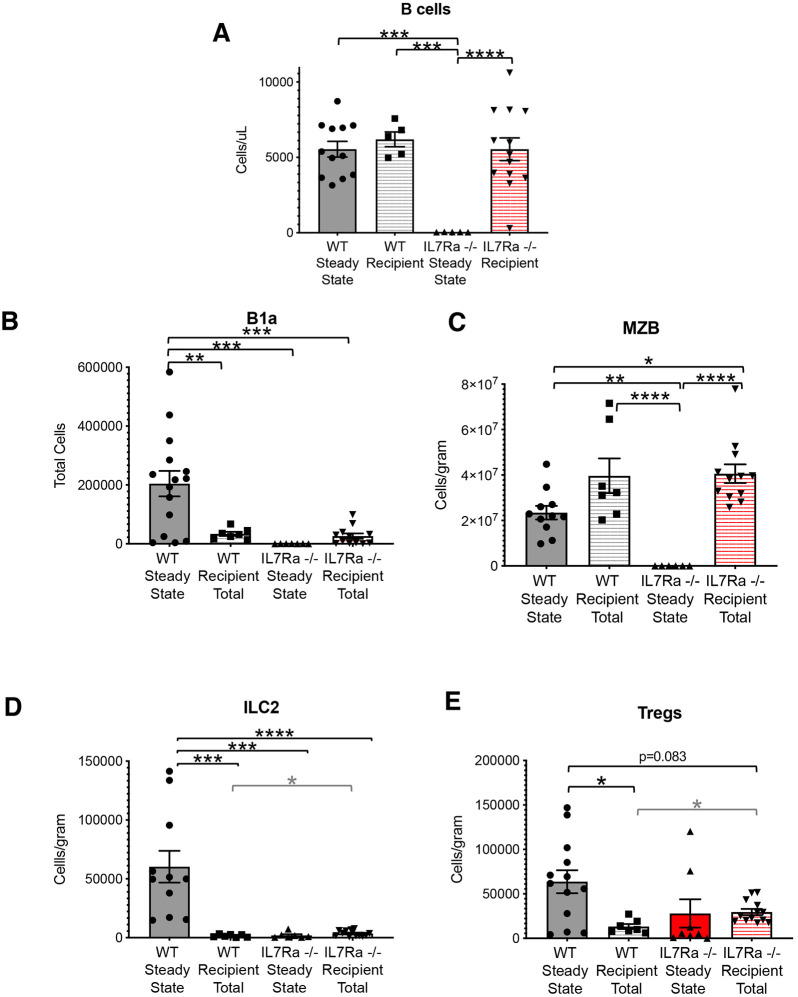
**WT HSCs transplanted into IL7Rα^−/−^ mice are not capable of reconstituting tissue-resident lymphocytes to steady-state numbers.** (A-E) Bar graphs comparing circulating B cells and TLCs at steady state (solid bars) to their reconstitution upon transplantation of WT HSCs (pattern bars). Cellularity of peripheral blood B cells (A), B1a cells (B), MZB (C), ILC2 (D) and Tregs (E) in WT or IL7Rα^−/−^ mice are compared with WT and IL7Rα^−/−^ mice reconstituted with WT HSCs. WT steady state *n*=11; WT recipient total *n*=7; IL7Rα^−/−^ steady state *n*=6; IL7Rα^−/−^ recipient total *n*=12. Differences were analyzed with one-way ANOVA: peripheral blood B cells *P*<0.0001; B1a cells *P*<0.0001; MZB *P*<0.0001; ILC2 *P*<0.0001; Tregs *P*=0.0134. Dunnett multi-parameter test: *P<0.05, **P<0.005, ***P<0.0005, ****P<0.00005. In gray are differences analyzed with unpaired two-tailed Student's *t*-test as reported in Fig. 4: *P<0.05.

The majority of TLCs are thought to arise pre/perinatally, with little contribution from adult progenitors. Although we observed enhanced reconstitution of some TLCs by adult WT HSCs in IL7Rα^−/−^ hosts, it remained unclear whether the lymphoid deficiencies observed in the IL7Rα^−/−^ mice (Fig. 2) were fully rescued by transplantation of adult WT HSCs. Therefore, we compared steady-state numbers of circulating lymphocytes and TLCs in both WT and IL7Rα^−/−^ mice (Fig. 2, solid bars) with total numbers after long-term hematopoietic reconstitution post-transplantation of WT HSCs (Fig. 4B′-E′). In WT and IL7Rα^−/−^ mice transplanted with WT HSCs, we found no significant difference in total peripheral blood B cells or T cells, or peritoneal B1b and B2 cells, compared with WT mice at steady state (Fig. 5A; Figs S2D, S1E,E′). Similarly, we observed no difference between total numbers of MZBs in WT mice at steady state and WT mice transplanted with WT HSCs (Fig. 5C, solid gray bar compared with gray lined bar). Therefore, WT HSCs were indeed capable of fully reconstituting these cells. In fact, although the difference in total MZBs was not significantly different between WT and IL7Rα^−/−^ recipients (Fig. 4C′), we observed significantly more MZBs in the IL7Rα^−/−^ recipient mice compared with WT steady state (Fig. 5C, solid gray bar compared with red lined bar). Interestingly, this was not the case for the other cell types examined. We observed significantly fewer B1a cells, ILC2s and Tregs in mice transplanted with WT HSCs compared with steady-state WT mice (Fig. 5B,D,E, solid gray bars compared with gray striped bars), consistent with reports by us and others of limited TLC potential of adult progenitors (Kikuchi and Kondo, 2006; Beaudin et al., 2016; Schneider et al., 2019). Despite the enhanced reconstitution of ILC2 and Tregs observed in the IL7Rα^−/−^ environment (Fig. 4D′-E′), we were surprised to find that this was not sufficient to return cell numbers to those observed in WT steady-state mice; rather, the total number of cells was not greater than the total numbers observed at steady state in the IL7Rα^−/−^ mice (Fig. 5D,E, solid red bars compared with red lined bars). Taken together, these data suggest that adult WT HSCs transplanted into IL7Rα^−/−^ mice are not fully capable of rescuing the impaired lymphoid phenotype caused by IL7Rα deletion and that there is differential requirement for IL7Rα on the development of tissue-resident B and T cells.

### Fetal HSCs outcompete adult HSCs in TLC reconstitution

**Fig. 6.**
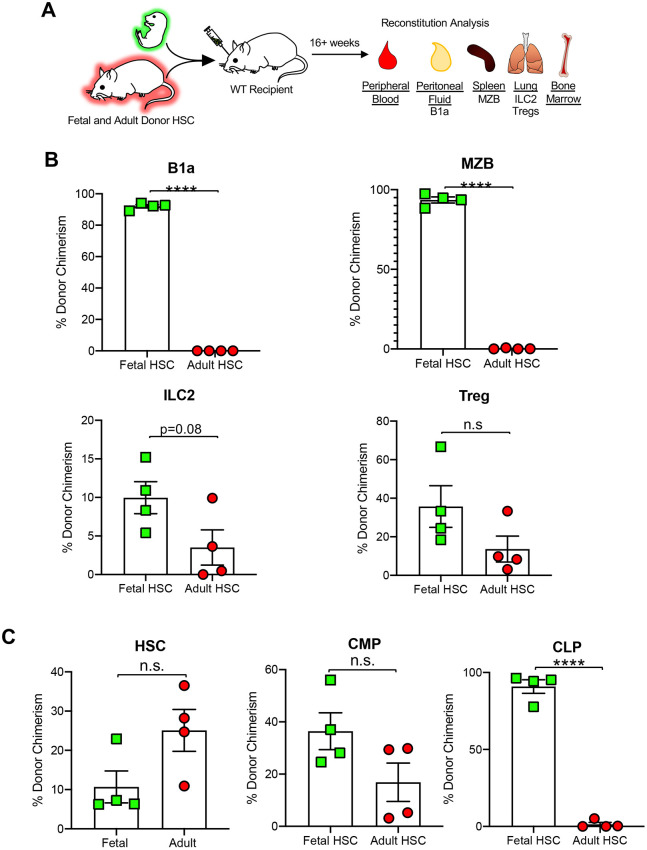
**Fetal HSCs reconstitute TLCs with greater efficiency compared with adult HSCs.** (A) Schematic of experimental design: 250 fetal HSCs and 250 adult HSCs from different fluorescent mice (KuO and UBC-GFP, respectively) were co-transplanted into sublethally irradiated WT recipients and donor contribution to hematopoietic cells was quantified in several organs 16 weeks post-transplantation. (B) Donor chimerism of TLCs was significantly greater by fetal HSCs compared with adult HSCs. Quantification of donor chimerism of peritoneal B1a cells, splenic MZBs, and lung ILC2s and Tregs. (C) Fetal and adult HSCs engrafted in the BM with similar efficiencies, but donor chimerism of fetal-derived CLPs was significantly greater than adult-derived CLPs. Quantification of donor chimerism of fetal and adult HSC, CMP and CLP in recipient BM. Fetal HSC *n*=4, adult HSC *n*=4, two independent experiments. Differences were analyzed with unpaired two-tailed Student's *t*-test: ****P<0.0001. n.s., not significant.

Our results in Fig. 3 clearly demonstrate that adult HSCs can contribute robustly to all four subsets of TLCs that we investigated. As referenced above, there is evidence for a fetal origin of some TLCs (Mold et al., 2010; Beaudin et al., 2016; Schneider et al., 2019; Azevedo Portilho et al., 2021) and the relative contribution of fetal and adult HSCs to TLCs is unclear. There is particular controversy over the capacity of adult HSCs to contribute to B1a cells (Beaudin and Forsberg, 2016), as studies have reported opposing findings (Montecino-Rodriguez et al., 2016; Ghosn et al., 2016; Kristiansen et al., 2016; Sawai et al., 2016). Therefore, we sought to determine the reconstitution capacity of both fetal and adult HSCs to generate TLCs. We performed competitive transplants of equal numbers of fetal and adult HSCs to allow for direct comparison of TLC reconstitution in the same host (Fig. 6A). We were surprised that fetal HSCs almost completely outcompeted adult HSCs, contributing significantly more to donor chimerism of B1a cells and MZBs (Fig. 6B) despite demonstrating reliable adult HSC reconstitution of B1a cells and MZB cells when transplanted without competing fetal HSCs (Fig. 3C,D). These findings were also observed for peritoneal B1b and B2 cells (Fig. S5A), and peripheral blood GM, B and T cells (Fig. S5B). Interestingly, ILC2 and Treg donor chimerism was not as drastically different between fetal and adult HSC compared with B1a and MZB (Fig. 6B). To determine whether the difference we observed in TLC reconstitution was due to HSC engraftment, we analyzed HSPCs in the bone marrow of recipient mice. We found no significant differences in donor chimerism of fetal-derived HSCs compared with adult derived HSCs, or common myeloid progenitors (CMPs) (Fig. 6C). In contrast, we did observe significantly greater donor chimerism of fetal-derived CLPs compared with adult-derived CLPs. These data suggest that fetal reconstitution of B1a and MZB might rely heavily on CLP-dependent pathways of differentiation, whereas adult reconstitution of ILC2 and Tregs may differentiate via alternate, CLP-independent (but IL7Rα-dependent) pathways, as suggested previously (Ghaedi et al., 2016).

## DISCUSSION

### Cell-intrinsic IL7Rα is required for differentiation of tissue-resident lymphoid cells

In this study, we aimed to expand our understanding on the roles of the cytokine receptors Flk2 and IL7Rα beyond traditional, circulating lymphocytes by investigating their roles in TLC development and reconstitution. Using lineage tracing, knockout mouse models and transplantation assays, our data demonstrated a clear role of IL7Rα in the differentiation of tissue-resident lymphoid cells across multiple tissues. B1a cells, MZBs, ILC2 and Tregs all displayed very high labeling in both the FlkSwitch and Il7rSwitch lineage tracing models; however, only IL7Rα^−/−^ resulted in severely impaired TLC development, whereas the effects of Flk2^−/−^ were minimal. Furthermore, IL7Rα^−/−^ HSCs were incapable of reconstituting TLCs in WT hosts, whereas WT HSCs were capable of TLC reconstitution in IL7Rα^−/−^ hosts, revealing a primarily cell-intrinsic requirement of IL7Rα for TLC differentiation. Interestingly, although adult HSCs were clearly capable of reconstituting all TLCs investigated, reconstitution of TLCs by fetal HSCs was notably greater compared with adult HSCs upon competitive transplantation.

### Flk2 is less essential for tissue-resident lymphocytes than for circulating lymphocytes

Based on the high labeling in the FlkSwitch model (Fig. 1) and our previous finding that multipotent, myeloid and lymphoid progenitors, and peripheral blood B cells were reduced in Flk2^−/−^ mice (Beaudin et al., 2014), we expected to find significantly reduced numbers of TLCs in Flk2-deficient mice. We were surprised that only MZBs were significantly reduced in the Flk2^−/−^ compared with WT mice (Fig. 2; Fig. S2A). We have also previously shown that multilineage reconstitution was impaired upon transplantation of Flk2^−/−^ HSCs in WT recipients, likely due to the significant decrease in HSC differentiation into Flk2^+^ multipotent progenitors (MPPs), and CLPs (Beaudin et al., 2014). Despite demonstrating that B1a cells, MZBs, ILC2 and Tregs differentiate via a Flk2-positive stage (Fig. 1), we were surprised that, amongst TLCs, only B1a and MZB reconstitution was impaired by the loss of Flk2 (Fig. 3). This may be because of a more stringent reliance on Flk2 for innate-like B cells rather than innate-like T cells, or reported CLP-independent differentiation of ILCs and T cells (Ghaedi et al., 2016). The notion of partial CLP-independence for ILC2 and Treg differentiation is also supported by the fetal and adult HSC reconstitution data, as we observed no significant differences in fetal and adult contribution to ILC2s and Tregs (Fig. 6B) despite a significant reduction in adult-derived CLPs (Fig. 6C). Interestingly, previous studies have shown that Tregs increase in number in response to the Flk2 ligand in mice and humans (McGee et al., 2010; Klein et al., 2013), making it all the more surprising that Tregs are the least affected by the loss of Flk2. These data suggest that IL7Rα is able to compensate for the loss of Flk2 in a cell-type specific manner but not vice versa, and therefore plays a more essential role in TLC reconstitution.

### Absolute cell quantification and competitive HSC transplantation revealed unexpected reconstitution capability

In all transplantation experiments, we employed an absolute cell quantification method previously developed in the lab (Boyer et al., 2019; Leung et al., 2019; Cool et al., 2020; Poscablo et al., 2021). This method was crucial to our interpretation of WT HSC reconstitution capability in IL7Rα^−/−^ recipients because it cannot be gleaned from percent donor chimerism alone. As the IL7Rα^−/−^ recipients were already devoid of any of the cells we were examining, donor-derived cells would be constituting 100% donor chimerism. By quantifying absolute cell numbers, we found that not only did WT HSCs generate TLCs in an IL7Rα^−/−^ recipient, but that the overall numbers of ILC2 and Tregs were greater in the IL7Rα^−/−^ recipients (Fig. 4). We suspect this is owing to the abundance of IL7 in the IL7Rα^−/−^ mice (Martin et al., 2017; Cool et al., 2020) and lack of host competition for IL7, which may in turn enhance the ILC2 and Treg reconstitution. Therefore, IL7Rα may have a cell non-autonomous role in regulating TLC subsets that rely more heavily on IL7 signaling by controlling IL7 levels (Martin et al., 2017; Cool et al., 2020). In addition, in our previous examination of eosinophil reconstitution in IL7Rα^−/−^ mice, we discovered that the concentrations of other cytokines involved in immune cell survival are different in IL7Rα^−/−^ compared with WT mice (Cool et al., 2020). Therefore, we speculate that differences in cytokine signals may play a significant role in the differences we observed in Fig. 4. Testing of a number of candidates did not uncover significant upregulation of lymphoid-promoting factors, leaving the previously documented increase in IL7 levels the sole current candidate for the increased reconstitution in IL7Rα^−/−^ mice over WT recipients.

We were also able to compare total TLCs reconstituted by WT HSCs to their total numbers *in situ* at steady state. With the exception of MZBs in the IL7Rα^−/−^ host, WT HSCs in either the WT host or the IL7Rα^−/−^ host did not fully rescue TLCs to WT steady-state levels (Fig. 5). This could conceivably be because we performed these transplants with adult HSCs and at least some types of TLCs are thought to originate primarily from fetal/neonatal progenitors. Recent work from Locksley and colleagues reported that ILC2s from the lung originate from perinatal progenitors (Schneider et al., 2019), and for decades it has remained controversial to what extent adult HSCs contribute to B1a cells. Here, we found robust B1a cell reconstitution from adult HSCs when transplanted alone (Fig. 3B), but this capacity was drastically overshadowed by fetal HSCs when transplanted competitively (Fig. 6B). Importantly, the fetal versus adult HSC competitiveness did not segregate by cell types often designated as fetal- versus adult-derived, as fetal HSCs also contributed significantly more to other, adult-derived peritoneal and circulating immune cells (Fig. S5). Mechanistically, this was not because of differences in HSC engraftment (Fig. 6C), but more likely related to greater proliferative and lymphoid capacity of fetal relative to adult HSCs, also manifested by significant differences in CLP reconstitution and a trend towards more fetal-derived CMPs (Fig. 6C). These results could be explained by the presence of developmentally restricted HSCs (drHSCs) within the fetal, but not adult, HSC compartment (Beaudin et al., 2016).

Although co-transplantation allowed us to more directly compare HSC reconstitution, our results show that competitive strategies may also obscure certain potential because one HSC type can outcompete the other in one or more lineages. This calls for close attention to the conditions of the assays used to claim reconstitution potential or lack thereof. The absolute quantification comparison may be a better indicator of the ability of adult HSCs to restore homeostasis, as these results revealed impairment in putative fetal-derived B1a, ILC2 and Tregs (Fig. 5B,D,E) but not adult-derived peritoneal or peripheral blood cells (Fig. 5A; Figs S1E,E′, S2D). These new perspectives will be valuable for more completely unraveling the cellular and molecular mechanisms that control TLC potential of fetal and adult HSCs; these clearly deserve further investigation, considering that HSCs are used in the clinic to treat a multitude of immune deficiencies.

### IL7Rα is required for both innate and adaptive immunity

**Fig. 7.**
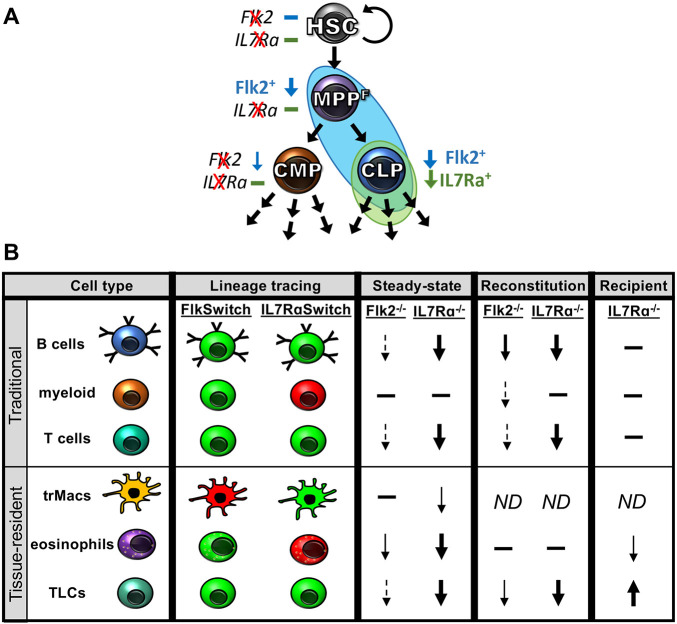
**Model summary of Flk2- and IL7R-mediated effects on hematopoiesis.** (A) The Flk2 and IL7R effects on downstream progeny are likely asserted at least in part by their expression on early hematopoietic progenitors, including multipotent progenitor cells (MPP^F^) and common lymphoid progenitors (CLPs). Flk2 expression on MPPs, CLPs and downstream lymphoid cells is indicated by blue Flk2^+^ text and by blue background. IL7R expression on CLPs and downstream lymphoid cells is indicated by green IL7R^+^ text and green background. The blue and green arrows indicate that the respective cell type is reduced upon deletion of either Flk2 or IL7R. (B) Differential effects of Flk2 and IL7R in the development and maintenance of hematopoietic cell types. Columns depict cell types, lineage tracing, steady-state analysis, HSC reconstitution of WT recipients and WT HSC transplantation into IL7R-deficient recipients. Rows depict six categories of immune cells: traditional B, T or myeloid cells (rows 1-3), or tissue-resident macrophages (trMacs), lung eosinophils, and lymphoid cells (TLCs) (rows 4-6). Results of Flk2 and IL7R lineage tracing are indicated in red (no history of expression) and green (differentiation occurred via cells expressing Flk2 or IL7R). Results of Flk2 and IL7R deletion on cell numbers is indicated by arrows pointing up (increase) or down (decrease); magnitude of change indicated by weight of arrows (dashed arrows, smallest change; thick arrow, largest change) no change is indicated by a horizontal bar; ND, no data.

Despite some TLCs displaying immune properties reminiscent of myeloid cells (Martin et al., 2001; Gunn and Brewer, 2006; Ha et al., 2006; Drake and Kita, 2014; Panda and Colonna, 2019), we demonstrated here that they nevertheless rely on main regulators of traditional circulating lymphoid cells (Fig. 7). The regulation of immune cell development by Flk2 and IL7R is highly complex, driven by partially overlapping expression and function of these receptors on hematopoietic progenitor cells (Fig. 7A) (Mackarehtschian et al., 1995; Christensen and Weissman, 2001; Schlenner et al., 2010; Boyer et al., 2011). In addition, we found that HSC competition can greatly influence the perceived ability to reconstitute hematopoiesis: when transplanted alone, adult HSCs provided robust TLC contribution (Fig. 3), which was almost completely overshadowed by fetal HSC-derived TLCs in competitive transplantations (Fig. 6). This may lead to reassessment of ‘fetal-derived’ cells and how to test and interpret the reconstitution potential of progenitor cells. Interestingly, IL7Rα regulation of TLC generation appeared to be predominantly cell intrinsic, similar to that of fetal-specified trMacs (Leung et al., 2019), but in contrast to the solely cell extrinsic role we discovered for IL7Rα in adult myeloid eosinophil regulation (Cool et al., 2020). The combination of cell intrinsic and extrinsic regulation by Flk2 and IL7R, and the dynamic secretion of their corresponding cytokines Flk2L and IL7 under different conditions, support a model of cell interdependence and complex, tissue-specific feedback (Fig. 7B) (from Figs 1-5, Figs S2,S3 and Beaudin et al., 2014; Leung et al., 2019; Cool et al., 2020). This interconnectedness can be further unraveled by future tissue-specific deletions of Flk2, Flk2L, IL7Ra, IL7 and/or other cytokines that selectively promote immune development. Collectively, these data indicate broad and temporally dynamic functional roles of both Flk2 and IL7Rα beyond the typical lymphoid ascribed roles.

## MATERIALS AND METHODS

### Mice

All animals were housed and bred in the Association for Assessment and Accreditation of Laboratory Animal Care International (AAALAC)-accredited vivarium at University of California, Santa Cruz (UCSC) and group housed in ventilated cages on a standard 12 h light/12 h dark cycle. All procedures were approved by the UCSC Institutional Animal Care and Use (IACUC) Committees (OLAW assurance A3859-01; USDA Registration 93-R-0439, customer number 9198). Il7r-Cre (Schlenner et al., 2010) and Flk2-Cre (Benz et al., 2008) mice, obtained under fully executed Material Transfer Agreements, were crossed to homozygous Rosa26^mTmG^ females (JAX Stock #007576, The Jackson Laboratory) (Muzumdar et al., 2007) to generate ‘switch’ lines, all on the C57BL/6 background. WT C57BL/6, Rosa26^mTmG^, UBC-GFP (JAX Stock #004353, The Jackson Laboratory) (Schaefer et al., 2001) and KuO (Hamanaka et al., 2013) mice were used for controls. FIDKO mice were generated by crossing Flk2^−/−^ (Mackarehtschian et al., 1995) and IL7Rα^−/−^ (Peschon et al., 1994) mice to homozygosity. Adult male and female mice (8-12 weeks old) were used randomly and indiscriminately, with the exception of the FlkSwitch line, in which only males were used because many female mice do not carry a Cre allele.

### Tissue and cell isolation

Mice were sacrificed by CO_2_ inhalation. Adult lung and spleen were isolated, weighed, then placed into 1.5 ml of digestion buffer [1× PBS(^+^/^+^) with 2% fetal bovine serum, 1 mg/ml (lung) or 2 mg/ml (spleen) collagenase IV (Gibco) with 100 U/ml DNase1] containing Calibrite APC-labeled (BD Biosciences) counting beads and manually disassociated into 2 mm×2 mm pieces using surgical scissors. The tissues were then incubated at 37°C for 1 h (lung) or 2 h (spleen). Following incubation, all tissues were passaged through 19 g then a 16 g needle 10 times, and then filtered through a 70 µM filter and 25 ml of cell staining buffer (1× PBS with 2% fetal bovine serum and 5 mM EDTA) was added to quench the digestion enzymes.

### Flow cytometry and cell analysis

Cell labeling was performed on ice in 1× PBS with 5 mM EDTA and 2% fetal bovine serum. Fluorescence-activated cell sorting (FACS) analysis was performed on a BD FACS Aria III (BD Biosciences) at UCSC, and analyzed using FlowJo (Treestar) (Boyer et al., 2019; Smith-Berdan et al., 2019; Martin et al., 2021; Poscablo et al., 2021). Cells were defined as follows: B1a=Lin^−^ (Ter119, CD11b, CD3) IgM^+^ CD5^+^ CD11b^mid^; MZB=Lin^−^ (CD3, CD4, CD8, Ter119, Gr1) B220^+^ IgM^+^ CD21^+^ CD23^−^; ILC2=Lin^−^ (CD3, CD4, CD5, CD8, NK1-1, CD19, Ter119, F4/80, FcεRIα) CD45^+^ KLRG1^+^ Sca1^+^ CD25^+^; Treg=Lin^−^ (Ter119, Gr1) CD3^+^ CD4^+^ TCRB^+^ CD25^+^ FOX-P3^+^.

### Transplantation assays

Transplantation assays were performed as previously described (Beaudin et al., 2014; Smith-Berdan et al., 2015; Ugarte et al., 2015; Beaudin et al., 2016). Briefly, double sorted HSCs were isolated from bone marrow of either WT (non-fluorescent, mTmG, UBC-GFP or KuO), Flk2^−/−^, Il7Rα^−/−^ or FIDKO 8-12 week old mice and from embryonic day (E)14.5 fetal livers. HSCs were defined as KLS (cKit^+^Lin^−^Sca1^+^), CD150^hi^Flk2^−^. Lineage markers were CD3, CD4, CD5, CD8, B220, Mac1, Gr1 and Ter119. Mac1 antibodies were excluded from the lineage cocktail when sorting fetal progenitors (Morrison et al., 1995). Flk2 could not be used to identify HSCs in Flk2^−/−^ and FIDKO HSCs and so they were sorted on KLS, CD150^hi^. Recipient mice aged 8-12 weeks were sublethally irradiated (750 rad, single dose) with a Faxitron CP-160. Under isofluorane-induced general anesthesia, sorted cells were transplanted retro-orbitally. Recipient mice were bled 4, 8, 12 and 16 weeks post-transplantation via tail vein and peripheral blood was analyzed for donor chimerism by means of fluorescence profiles and antibodies to lineage markers (Table S1). Long-term multilineage reconstitution was defined as chimerism in both the lymphoid and myeloid lineages of >0.1% at 16 weeks post-transplantation and only mice that displayed long-term reconstitution were used for post-transplantation analysis.

### Absolute cell number quantification

A known volume of peripheral blood was mixed with an antibody solution, (PBS, 5 mM EDTA and 2% fetal bovine serum) containing a known quantity of Calibrite APC beads before flow cytometry analysis (Boyer et al., 2019; Cool et al., 2020; Rajendiran et al., 2020; Poscablo et al., 2021). For tissues, a known quantity of beads was added at the very beginning of tissue preparation before antibody staining and analysis. The number of beads counted by flow cytometry was used to calculate the number of mature cells per ml of blood or within each tissue.

### Quantification and statistical analysis

Number of experiments, *n*, and what *n* represents can be found in the legend for each figure. Power analysis to determine the minimum size of experimental groups was performed with a confidence level of 0.95 and desired power of 0.8 using an online tool (https://epitools.ausvet.com.au/). Statistical significance was determined by two-tailed unpaired Student's *t*-test when comparing two groups and by one-way ANOVA and Dunnett multi-parameter test when comparing across multiple groups. In Figs 2-4, 6 and Figs S2A-C′, S3-S5, all comparisons are being made to WT controls, and not across all groups despite being graphed together. All data are shown as mean±s.e.m. representing at least two independent experiments.

## Supplementary Material

Supplementary information

Reviewer comments

## References

[DEV200139C2] Azevedo Portilho, N., Scarfò, R., Bertesago, E., Ismailoglu, I., Kyba, M., Kobayashi, M., Ditadi, A. and Yoshimoto, M. (2021). B1 lymphocytes develop independently of Notch signaling during mouse embryonic development. *Development* 148, dev199373. 10.1242/dev.199373 34370006 PMC8380455

[DEV200139C3] Bayer, A. L., Lee, J. Y., De La Barrera, A., Surh, C. D. and Malek, T. R. (2008). A function for IL-7R for CD4+CD25+Foxp3+T regulatory cells. *J. Immunol.* 181, 225-234. 10.4049/jimmunol.181.1.225 18566388 PMC2601574

[DEV200139C4] Beaudin, A. E. and Forsberg, E. C. (2016). To B1a or not to B1a: do hematopoietic stem cells contribute to tissue-resident immune cells? *Blood* 128, 2765-2769. 10.1182/blood-2016-10-697813 27799163 PMC5159701

[DEV200139C5] Beaudin, A. E., Boyer, S. W. and Forsberg, E. C. (2014). Flk2/Flt3 promotes both myeloid and lymphoid development by expanding non–self-renewing multipotent hematopoietic progenitor cells. *Exp. Hematol.*, 42, 218-229.e4. 10.1016/j.exphem.2013.11.013 24333663 PMC4047989

[DEV200139C6] Beaudin, A. E., Boyer, S. W., Perez-Cunningham, J., Hernandez, G. E., Derderian, S. C., Jujjavarapu, C., Aaserude, E., Mackenzie, T. and Forsberg, E. C. (2016). A transient developmental hematopoietic stem cell gives rise to innate-like B and T cells. *Cell Stem Cell* 19, 768-783. 10.1016/j.stem.2016.08.013 27666010 PMC5524382

[DEV200139C7] Benz, C., Martins, V. C., Radtke, F. and Bleul, C. C. (2008). The stream of precursors that colonizes the thymus proceeds selectively through the early T lineage precursor stage of T cell development. *J. Exp. Med.* 205, 1187-1199. 10.1084/jem.20072168 18458114 PMC2373849

[DEV200139C64] Blériot, C., Chakarov, S. and Ginhoux, F. (2020) Determinants of resident tissue macrophage identity and function. *Immunity* 52, 957-970. 10.1016/j.immuni.2020.05.014 32553181

[DEV200139C8] Bonilla, F. A. and Oettgen, H. C. (2010). Adaptive immunity. *J. Allergy Clin. Immunol.* 125 Suppl. 2, S33-S40. 10.1016/j.jaci.2009.09.017 20061006

[DEV200139C9] Boyer, S. W., Schroeder, A. V., Smith-Berdan, S. and Forsberg, E. C. (2011). All hematopoietic cells develop from hematopoietic stem cells through Flk2/Flt3-positive progenitor cells. *Cell Stem Cell* 9, 64-73. 10.1016/j.stem.2011.04.021 21726834 PMC4103692

[DEV200139C10] Boyer, S. W., Beaudin, A. E. and Forsberg, E. C. (2012). Mapping differentiation pathways from hematopoietic stem cells using Flk2/Flt3 lineage tracing. *Cell cycle* 11, 3180-3188. 10.4161/cc.21279 22895180 PMC3466517

[DEV200139C11] Boyer, S. W., Rajendiran, S., Beaudin, A. E., Smith-Berdan, S., Muthuswamy, P. K., Perez-Cunningham, J., Martin, E. W., Cheung, C., Tsang, H., Landon, M. et al. (2019). Clonal and quantitative In Vivo assessment of hematopoietic stem cell differentiation reveals strong erythroid potential of multipotent cells. *Stem Cell Reports* 12, 801-815. 10.1016/j.stemcr.2019.02.007 30905737 PMC6450035

[DEV200139C12] Chou, C. and Li, M. O. (2018). Tissue-resident lymphocytes across innate and adaptive lineages. *Front. Immunol.* 9, 2104. 10.3389/fimmu.2018.02104 30298068 PMC6160555

[DEV200139C13] Christensen, J. L. and Weissman, I. L. (2001). Flk-2 is a marker in hematopoietic stem cell differentiation: a simple method to isolate long-term stem cells. *Proc. Natl. Acad. Sci. USA* 98, 14541-14546. 10.1073/pnas.261562798 11724967 PMC64718

[DEV200139C14] Cool, T. and Forsberg, E. C. (2019). Chasing mavericks: the quest for defining developmental waves of hematopoiesis. *Curr. Top. Dev. Biol.* 132, 1-29. 10.1016/bs.ctdb.2019.01.001 30797507 PMC9107351

[DEV200139C15] Cool, T., Worthington, A., Poscablo, D., Hussaini, A. and Forsberg, E. C. (2020). Interleukin 7 receptor is required for myeloid cell homeostasis and reconstitution by hematopoietic stem cells. *Exp. Hematol.* 90, 39-45.e3. 10.1016/j.exphem.2020.09.001 32916215 PMC7951140

[DEV200139C16] Davies, L. C., Jenkins, S. J., Allen, J. E. and Taylor, P. R. (2013). Tissue-resident macrophages. *Nat. Immunol.* 14, 986-995. 10.1038/ni.2705 24048120 PMC4045180

[DEV200139C17] Drake, L. Y. and Kita, H. (2014). Group 2 innate lymphoid cells in the lung. *Adv. Immunol.* 124, 1-16. 10.1016/B978-0-12-800147-9.00001-7 25175771 PMC4449733

[DEV200139C18] Ghosn, E. E. B., Yamamoto, R., Hamanaka, S., Yang, Y., Herzenberg, L. A., Nakauchi, H. and Herzenberg, L. A. (2012). Distinct B-cell lineage commitment distinguishes adult bone marrow hematopoietic stem cells. *Proc. Natl. Acad. Sci. U.S.A.* 109, 5394-5398. 10.1073/pnas.1121632109 22431624 PMC3325648

[DEV200139C19] Ghosn, E. E. B., Waters, J., Phillips, M., Yamamoto, R., Long, B. R., Yang, Y., Gerstein, R., Stoddart, C. A., Nakauchi, H. and Herzenberg, L. A. (2016). Fetal hematopoietic stem cell transplantation fails to fully regenerate the B-Lymphocyte compartment. *Stem Cell Reports* 6, 137-149. 10.1016/j.stemcr.2015.11.011 26724903 PMC4720028

[DEV200139C20] Epelman, S., Lavine, K. J., Beaudin, A. E., Sojka, D. K., Carrero, J. A., Calderon, B., Brija, T., Gautier, E. L., Ivanov, S., Satpathy, A. T. et al. (2014). Embryonic and adult-derived resident cardiac macrophages are maintained through distinct mechanisms at steady state and during inflammation. *Immunity* 40, 91-104. 10.1016/j.immuni.2013.11.019 24439267 PMC3923301

[DEV200139C21] Ghaedi, M., Steer, C. A., Martinez-Gonzalez, I., Halim, T. Y. F., Abraham, N. and Takei, F. (2016). Common-lymphoid-progenitor-independent pathways of innate and T lymphocyte development. *Cell Rep.* 15, 471-480. 10.1016/j.celrep.2016.03.039 27068476

[DEV200139C22] Guimond, M., Veenstra, R. G., Grindler, D. J., Zhang, H., Cui, Y., Murphy, R. D., Kim, S. Y., Na, R., Hennighausen, L., Kurtulus, S. et al. (2009). Interleukin 7 signaling in dendritic cells regulates the homeostatic proliferation and niche size of CD4+ T cells. *Nat. Immunol.* 10, 149-157. 10.1038/ni.1695 19136960 PMC2713006

[DEV200139C23] Gunn, K. E. and Brewer, J. W. (2006). Evidence that marginal zone b cells possess an enhanced secretory apparatus and exhibit superior secretory activity. *J Immunol.* 177, 3791-3798. 10.4049/jimmunol.177.6.3791 16951340

[DEV200139C24] Ha, S. A., Tsuji, M., Suzuki, K., Meek, B., Yasuda, N., Kaisho, T. and Fagarasan, S. (2006). Regulation of B1 cell migration by signals through Toll-like receptors. *J. Exp. Med.* 203, 2541-2550. 10.1084/jem.20061041 17060475 PMC2118139

[DEV200139C25] Hamanaka, S., Ooehara, J., Morita, Y., Ema, H., Takahashi, S., Miyawaki, A., Otsu, M., Yamaguchi, T., Onodera, M. and Nakauchi, H. (2013). Generation of transgenic mouse line expressing Kusabira Orange throughout body, including erythrocytes, by random segregation of provirus method. *Biochem. Biophys. Res. Commun.* 435, 586-591. 10.1016/j.bbrc.2013.05.017 23685154

[DEV200139C26] Hashimoto, D., Chow, A., Noizat, C., Teo, P., Beasley, M. B., Leboeuf, M., Becker, C. D., See, P., Price, J., Lucas, D. et al. (2013). Tissue-resident macrophages self-maintain locally throughout adult life with minimal contribution from circulating monocytes. *Immunity* 38, 792-804. 10.1016/j.immuni.2013.04.004 23601688 PMC3853406

[DEV200139C27] Hesslein, D. G. T., Yang, S. Y. and Schatz, D. G. (2006). Origins of peripheral B cells in IL-7 receptor-deficient mice. *Mol. Immunol.* 43, 326-334. 10.1016/j.molimm.2005.02.010 16310046

[DEV200139C63] Hoeffel, G., Chen, J., Lavin, Y. , Low, D., Almeida, F. F., See, P., Beaudin, A. E., Lum, J., Low, I., Forsberg, E. C. et al. (2015). C-Myb^+^ erythro-myeloid progenitor-derived fetal monocytes give rise to adult tissue-resident macrophages. *Immunity* 42, 665-678. 10.1016/j.immuni.2015.03.011 25902481 PMC4545768

[DEV200139C28] Jensen, C. T., Kharazi, S., BöIers, C., Cheng, M., Lübking, A., Sitnicka, E. and Jacobsen, S. E. W. (2008). FLT3 ligand and not TSLP is the key regulator of IL-7–independent B-1 and B-2 B lymphopoiesis. *Blood* 112, 2297-2304. 10.1182/blood-2008-04-150508 18566323

[DEV200139C29] Kadel, S., Ainsua-Enrich, E., Hatipoglu, I., Turner, S., Singh, S., Khan, S. and Kovats, S. (2018). A major population of functional KLRG1 - ILC2s in female lungs contributes to a sex bias in ILC2 numbers. *ImmunoHorizons* 2, 74-86. 10.4049/immunohorizons.1800008 29568816 PMC5860819

[DEV200139C30] Kantor, A. B. and Herzenberg, L. A. (1993). Origin of murine B cell lineages. *Annu. Rev. Immunol.* 11, 501-538. 10.1146/annurev.iy.11.040193.002441 8476571

[DEV200139C31] Kikuchi, K. and Kondo, M. (2006). Developmental switch of mouse hematopoietic stem cells from fetal to adult type occurs in bone marrow after birth. *Proc. Natl. Acad. Sci. U.S.A.* 103, 17852-17857. 10.1073/pnas.0603368103 17090683 PMC1693836

[DEV200139C32] Kikuchi, K., Kasai, H., Watanabe, A., Lai, A. Y. and Kondo, M. (2008). IL-7 specifies B cell fate at the CLP to pre-proB transition stage by maintaining EBF expression. *J. Immunol.* 181, 383-392. 10.4049/jimmunol.181.1.383 18566404 PMC2586338

[DEV200139C33] Klein, O., Ebert, L. M., Zanker, D., Woods, K., Tan, B. S., Fucikova, J., Behren, A., Davis, I. D., Maraskovsky, E., Chen, W. et al. (2013). Flt3 ligand expands CD4+FoxP3+regulatory T cells in human subjects. *Eur. J. Immunol.* 43, 533-539. 10.1002/eji.201242603 23124877

[DEV200139C34] Kristiansen, T. A., Jaensson Gyllenbäck, E., Zriwil, A., Björklund, T., Daniel, J. A., Sitnicka, E., Soneji, S., Bryder, D. and Yuan, J. (2016). Cellular barcoding links B-1a B cell potential to a fetal hematopoietic stem cell state at the single-cell level. *Immunity* 45, 346-357. 10.1016/j.immuni.2016.07.014 27533015

[DEV200139C35] Leung, G. A., Cool, T., Valencia, C. H., Worthington, A., Beaudin, A. E. and Forsberg, E. C. (2019). The lymphoid-associated interleukin 7 receptor (IL7R) regulates tissue-resident macrophage development. *Development* 146, dev176180. 10.1242/dev.176180 31332039 PMC6679362

[DEV200139C36] Mackarehtschian, K., Hardin, J. D., Moore, K. A., Boast, S., Goff, S. P. and Lemischka, I. R. (1995). Targeted disruption of the flk2/flt3 gene leads to deficiencies in primitive hematopoietic progenitors. *Immunity* 3, 147-161. 10.1016/1074-7613(95)90167-1 7621074

[DEV200139C37] Martin, F., Oliver, A. M. and Kearney, J. F. (2001). Marginal zone and B1 B cells unite in the early response against T-independent blood-borne particulate antigens. *Immunity* 14, 617-629. 10.1016/S1074-7613(01)00129-7 11371363

[DEV200139C38] Martin, C. E., Spasova, D. S., Frimpong-Boateng, K., Kim, H.-O., Lee, M., Kim, K. S. and Surh, C. D. (2017). Interleukin-7 availability is maintained by a hematopoietic cytokine sink comprising innate lymphoid cells and T cells. *Immunity* 47, 171-182.e4. 10.1016/j.immuni.2017.07.005 28723549

[DEV200139C39] Martin, E. W., Krietsch, J., Reggiardo, R. E., Sousae, R., Kim, D. H. and Forsberg, E. C. (2021). Chromatin accessibility maps provide evidence of multilineage gene priming in hematopoietic stem cells. *Epigenetics Chromatin* 14, 2. 10.1186/s13072-020-00377-1 33407811 PMC7789351

[DEV200139C65] Martin, P. and Gurevich, D. B. (2021). Macrophage regulation of angiogenesis in health and disease. *Semin. Cell Dev. Biol*. 119, 101-110. 10.1016/j.semcdb.2021.06.010 34330619

[DEV200139C40] McGee, H. S., Edwan, J. H. and Agrawal, D. K. (2010). Flt3-L increases CD4+CD25+Foxp3+ICOS+cells in the lungs of cockroach-sensitized and -challenged mice. *Am. J. Respir. Cell Mol. Biol.* 42, 331-340. 10.1165/rcmb.2008-0397OC 19448155 PMC2830405

[DEV200139C41] Mold, J. E., Venkatasubrahmanyam, S., Burt, T. D., Michaã“Lsson, J., Rivera, J. M., Galkina, S. A., Weinberg, K., Stoddart, C. A. and Mccune, J. M. (2010). Fetal and adult hematopoietic stem cells give rise to distinct T cell lineages in humans. *Science* 330, 1695-1699. 10.1126/science.1196509 21164017 PMC3276679

[DEV200139C42] Montecino-Rodriguez, E., Fice, M., Casero, D., Berent-Maoz, B., Barber, C. L. and Dorshkind, K. (2016). Distinct genetic networks orchestrate the emergence of specific waves of fetal and adult B-1 and B-2 development. *Immunity* 45, 527-539. 10.1016/j.immuni.2016.07.012 27566938 PMC5033716

[DEV200139C43] Morrison, S. J., Hemmati, H. D., Wandycz, A. M. and Weissman, I. L. (1995). The purification and characterization of fetal liver hematopoietic stem cells. *Proc. Natl. Acad. Sci. U.S.A.* 92, 10302-10306. 10.1073/pnas.92.22.10302 7479772 PMC40784

[DEV200139C44] Muzumdar, M. D., Tasic, B., Miyamichi, K., Li, L. and Luo, L. (2007). A global double-fluorescent Cre reporter mouse. *Genesis (New York, N.Y.: 2000)* 45, 593-605. 10.1002/dvg.20335 17868096

[DEV200139C45] Netea, M. G., Quintin, J. and Van Der Meer, J. W. M. (2011). Trained immunity: a memory for innate host defense. *Cell Host Microbe* 9, 355-361. 10.1016/j.chom.2011.04.006 21575907

[DEV200139C46] Osborne, L. C., Patton, D. T., Seo, J. H. and Abraham, N. (2011). Elevated IL-7 availability does not account for T cell proliferation in moderate lymphopenia. *J. Immunol.* 186, 1981-1988. 10.4049/jimmunol.1002224 21239710

[DEV200139C47] Panda, S. K. and Colonna, M. (2019). Innate lymphoid cells in mucosal immunity. *Front. Immunol.* 10, 1-13. 10.3389/fimmu.2019.00861 31134050 PMC6515929

[DEV200139C48] Patton, D. T., Plumb, A. W., Redpath, S. A., Osborne, L. C., Perona-Wright, G. and Abraham, N. (2014). The development and survival but not function of follicular B cells is dependent on IL-7Rα Tyr449 signaling. *PLoS ONE* 9, e88771. 10.1371/journal.pone.0088771 24551160 PMC3923819

[DEV200139C49] Peschon, J. J., Morrissey, P. J., Grabstein, K. H., Ramsdell, F. J., Maraskovsky, E., Gliniak, B. C., Park, L. S., Ziegler, S. F., Williams, D. E., Ware, C. B. et al. (1994). Early lymphocyte expansion is severely impaired in interleukin 7 receptor-deficient mice. *J. Exp. Med.* 180, 1955-1960. 10.1084/jem.180.5.1955 7964471 PMC2191751

[DEV200139C50] Poscablo, D. M., Worthington, A. K., Smith-Berdan, S. and Forsberg, E. C. (2021). Megakaryocyte progenitor cell function is enhanced upon aging despite the functional decline of aged hematopoietic stem cells. *Stem Cell Reports* 16, 1598-1613. 10.1016/j.stemcr.2021.04.016 34019813 PMC8190594

[DEV200139C51] Rajendiran, S., Boyer, S. W. and Forsberg, E. C. (2020). A quantitative hematopoietic stem cell reconstitution protocol: Accounting for recipient variability, tissue distribution and cell half-lives. *Stem Cell Res.* 50, 102145. 10.1016/j.scr.2020.102145 33486300 PMC8239062

[DEV200139C52] Robinette, M. L., Bando, J. K., Song, W., Ulland, T. K., Gilfillan, S. and Colonna, M. (2017). IL-15 sustains IL-7R-independent ILC2 and ILC3 development. *Nat. Commun.* 8, 14601. 10.1038/ncomms14601 28361874 PMC5380969

[DEV200139C53] Sawai, C. M., Babovic, S., Upadhaya, S., Knapp, D. J. H. F., Lavin, Y., Lau, C. M., Goloborodko, A., Feng, J., Fujisaki, J., Ding, L. et al. (2016). Hematopoietic stem cells are the major source of multilineage hematopoiesis in adult animals. *Immunity* 45, 597-609. 10.1016/j.immuni.2016.08.007 27590115 PMC5054720

[DEV200139C54] Schaefer, B. C., Schaefer, M. L., Kappler, J. W., Marrack, P. and Kedl, R. M. (2001). Observation of antigen-dependent CD8+T-cell/dendritic cell interactions in vivo. *Cell. Immunol.* 214, 110-122. 10.1006/cimm.2001.1895 12088410

[DEV200139C55] Schlenner, S. M., Madan, V., Busch, K., Tietz, A., Läufle, C., Costa, C., Blum, C., Fehling, H. J. and Rodewald, H.-R. (2010). Fate mapping reveals separate origins of T cells and myeloid lineages in the thymus. *Immunity* 32, 426-436. 10.1016/j.immuni.2010.03.005 20303297

[DEV200139C56] Schluns, K. S., Kieper, W. C., Jameson, S. C. and Lefrançois, L. (2000). Interleukin-7 mediates the homeostasis of naïve and memory CD8 T cells in vivo. *Nat. Immunol.* 1, 426-432. 10.1038/80868 11062503

[DEV200139C57] Schneider, C., Lee, J., Koga, S., Ricardo-Gonzalez, R. R., Nussbaum, J. C., Smith, L. K., Villeda, S. A., Liang, H.-E. and Locksley, R. M. (2019). Tissue-resident group 2 innate lymphoid cells differentiate by layered ontogeny and in situ perinatal priming. *Immunity* 50, 1425-1438.e5. 10.1016/j.immuni.2019.04.019 31128962 PMC6645687

[DEV200139C58] Sitnicka, E., Buza-Vidas, N., Ahlenius, H., Cilio, C. M., Gekas, C., Nygren, J. M., Månsson, R., Cheng, M., Jensen, C. T., Svensson, M. et al. (2007). Critical role of FLT3 ligand in IL-7 receptor-independent T lymphopoiesis and regulation of lymphoid-primed multipotent progenitors. *Blood* 110, 2955-2964. 10.1182/blood-2006-10-054726 17540845

[DEV200139C59] Smith-Berdan, S., Nguyen, A., Hong, M. A. and Forsberg, E. C. (2015). ROBO4-mediated vascular integrity regulates the directionality of hematopoietic stem cell trafficking. *Stem Cell Reports* 4, 255-268. 10.1016/j.stemcr.2014.12.013 25640759 PMC4325232

[DEV200139C60] Smith-Berdan, S., Bercasio, A., Rajendiran, S. and Forsberg, E. C. (2019). Viagra enables efficient, single-day hematopoietic stem cell mobilization. *Stem Cell Reports* 13, 787-792. 10.1016/j.stemcr.2019.09.004 31607567 PMC6895718

[DEV200139C61] Ugarte, F., Sousae, R., Cinquin, B., Martin, E. W., Krietsch, J., Sanchez, G., Inman, M., Tsang, H., Warr, M., Passegué, E. et al. (2015). Progressive chromatin condensation and H3K9 methylation regulate the differentiation of embryonic and hematopoietic stem cells. *Stem Cell Reports* 5, 728-740. 10.1016/j.stemcr.2015.09.009 26489895 PMC4649257

[DEV200139C62] Zhang, C. C. and Lodish, H. F. (2008). Cytokines regulating hematopoietic stem cell function. *Curr. Opin Hematol.* 15, 307-311. 10.1097/MOH.0b013e3283007db5 18536567 PMC2677548

