# Supplementary material for: IL7Rα, but not Flk2, is required for hematopoietic stem cell reconstitution of tissue-resident lymphoid cells
Source: Development. 2022 Jan 24;149(8):dev200139. doi: 10.1242/dev.200139 (PMC8917444; doi:10.1242/dev.200139)
Supplement: Supplementary information [file develop-149-200139-s1.pdf]

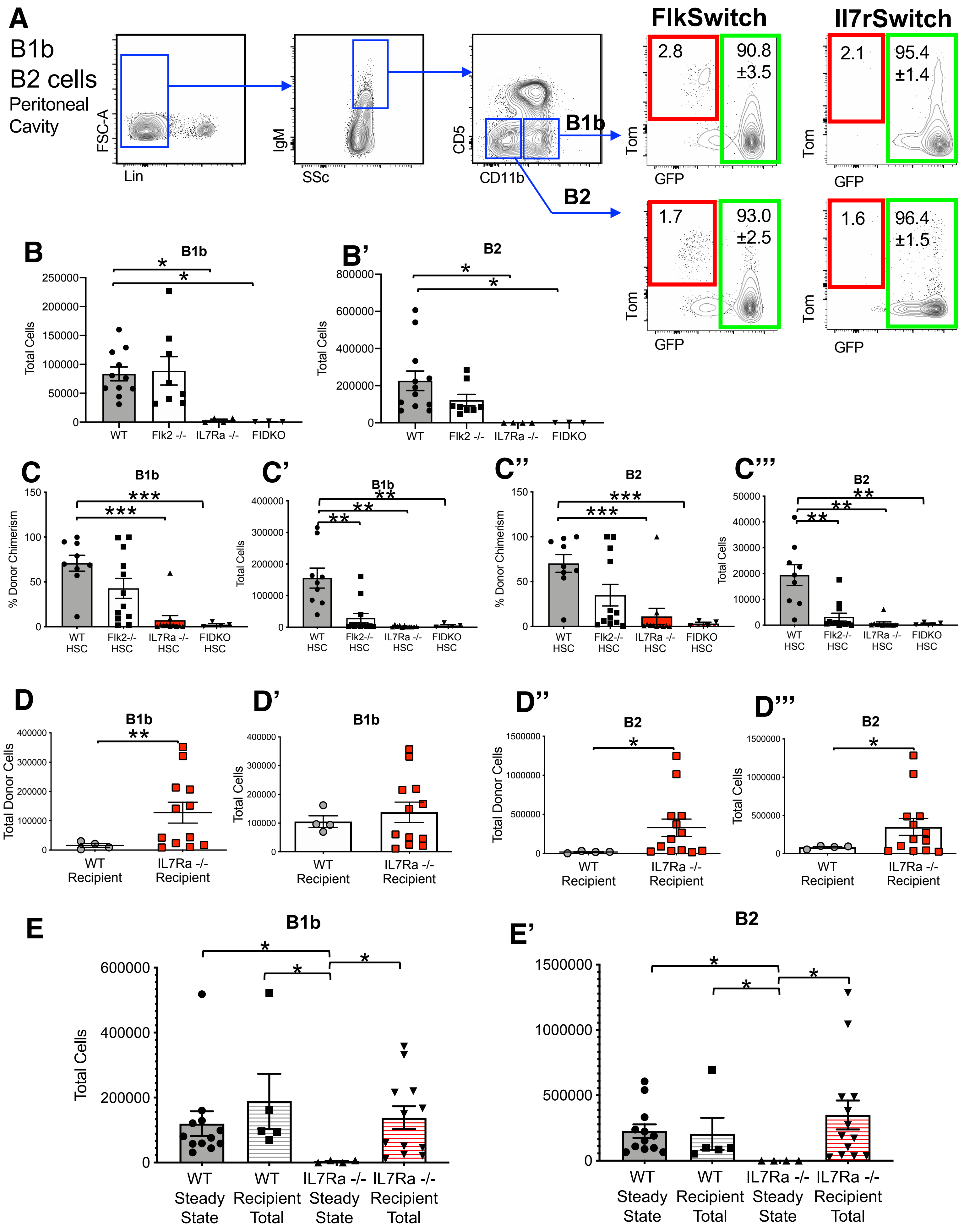

Development • Supplementary information

Fig. S1. B1b and B2 cells from the peritoneal cavity behave like traditional, circulating B-cells.

A) B1b ($Lin^-IgM^+CD5^-CD11b^{mid}$) and B2 ($Lin^-IgM^+CD5^-CD11b^-$) cells from the peritoneal cavity were highly labeled in both FlkSwitch and IL7rSwitch lineage tracing models. Representative flow cytometric analysis of reporter expression.

B) B1b and **B')** B2 cells were significantly reduced in $IL7R\alpha^{-/-}$ and FIDKO mice compared to WT. Quantification of total cell numbers in the peritoneal cavity. Data are representative of 4-5 mice per cohort in three independent experiments.

C, C'') Donor Chimerism of B1b and B2 cells was significantly reduced from $IL7R\alpha^{-/-}$ (red bars) and FIDKO (red square bars) donor HSCs compared to WT (grey bars) donor HSCs. Quantification of donor chimerism of B1b and B2 cells respectively.

C', C''') Cellularity of B1b and B2 cells was significantly reduced from $Flk2^{-/-}$ (white bars), $IL7R\alpha^{-/-}$ (red bars) and FIDKO (red square bars) donor HSCs compared to WT (grey bars) donor HSCs. Quantification of total B1b and B2 cells. WT n=9, $Flk2^{-/-}$ n=11, $IL7R\alpha^{-/-}$ n=7, FIDKO n=4, representing a minimum of four independent experiments. **D, D'')** Significantly greater donor-derived B1b and B2 cells in an $IL7R\alpha^{-/-}$ recipient (red squares) compared to a WT recipient (grey dots). Quantification of donor-derived cells of B1b and B2 cells, respectively.

D', D''') Significantly greater total B2 cells in an $IL7R\alpha^{-/-}$ recipient (red squared) compared to a WT recipient (grey dots). Quantification of total cells (donor+host) of B1b and B2, respectively. WT recipient n=5, $IL7R\alpha^{-/-}$ recipient n=13, representing four independent experiments.

E-E') Bar graph comparing circulating B cells and TLCs cells at steady state (solid bars) to their reconstitution upon transplantation of WT HSCs (pattern bars). Cellularity of B1b and B2 in WT or $IL7R\alpha^{-/-}$ mice are compared to WT and $IL7R\alpha^{-/-}$ mice reconstituted with WT HSCs. Differences in B-D) were analyzed with two-tailed Student t test; *, $P<0.05$; **, $P< 0.005$; ***, $P<0.0005$. Differences in E-E') were analyzed with one-way ANOVA; B1b $p=0.0107$ and B2 $p=0.0129$ and Dunnett multi-parameter test; *, $P<0.05$; **, $P< 0.005$; ***, $P<0.0005$.

Development • Supplementary information

Development: doi:10.1242/dev.200139: Supplementary information

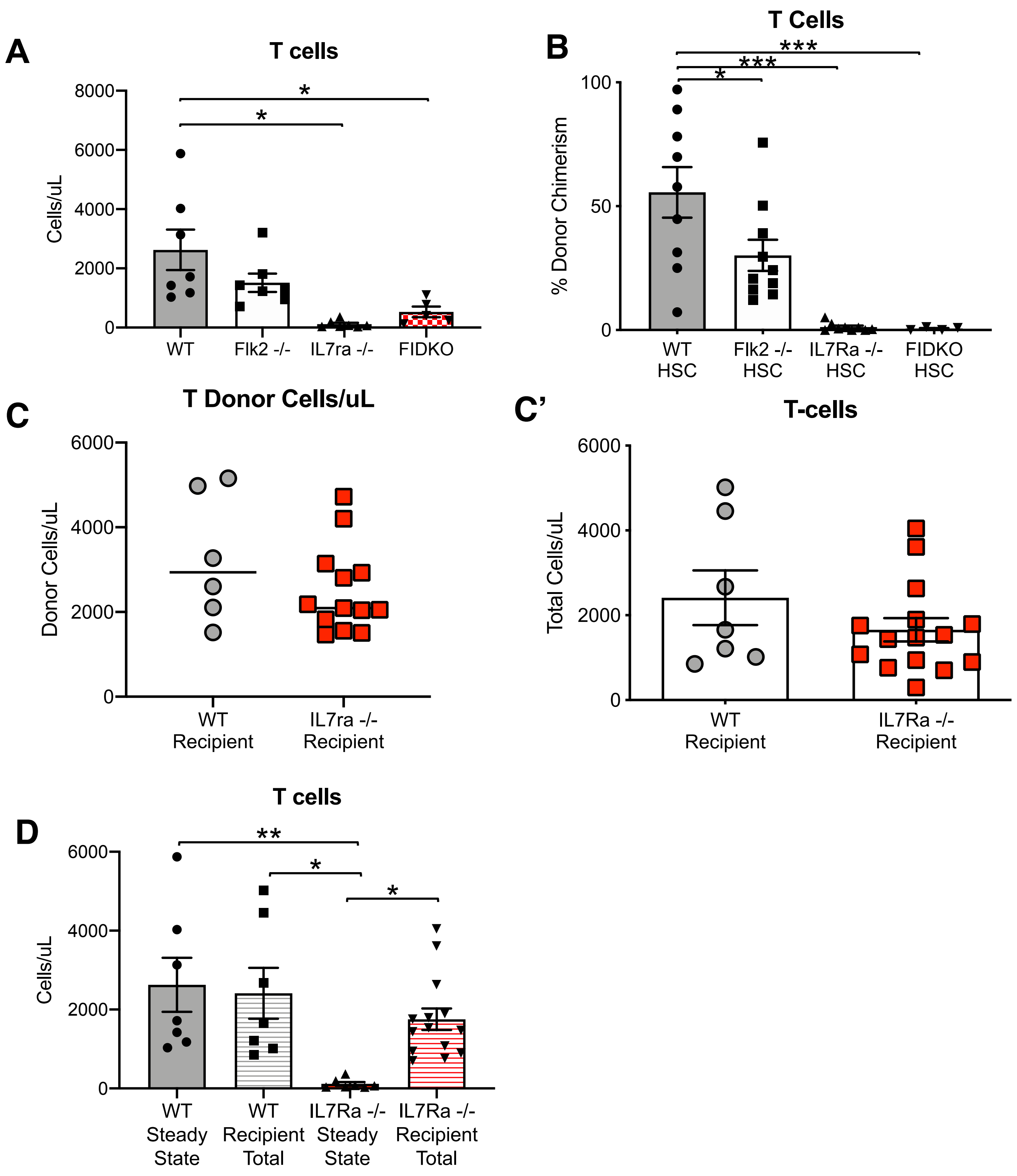

Development • Supplementary information

Fig. S2. CD3+ T cells in peripheral blood require IL7Rα.

A) Numbers of “traditional” CD3+ T cells in the peripheral blood of 8 to 12 week old WT, Flk2$^{-/-}$, IL7Rα$^{-/-}$ and FIDKO mice. WT (grey), Flk2$^{-/-}$ (white), IL7Rα$^{-/-}$ (red) and Flk2$^{-/-}$/IL7Rα$^{-/-}$ double knockout (FIDKO; red/white). Data from the same mice as in Figure 2.

B) 500 WT, Flk2$^{-/-}$, IL7Rα$^{-/-}$ or FIDKO HSCs were transplanted into sublethally irradiated fluorescent WT recipients (mTmG or UBC-GFP) and donor chimerism of peripheral blood CD3 + T cells was quantified 16 weeks post transplantation. Reconstitution of T cells was significantly impaired upon transplantation of IL7Rα$^{-/-}$ (red bars) and FIDKO HSCs (red/white bars) compared to WT HSCs (grey bars). Data from the same mice as in Figure 3.

C-C’). 500 fluorescent WT HSCs (UBC-GFP), were transplanted into sublethally irradiated non-fluorescent WT or IL7Rα$^{-/-}$ recipients. Donor contribution to peripheral blood CD3+ T cells was quantified 16 weeks post-transplantation. **C)** Donor-derived and **C’)** total T cells were not changed in an IL7Rα$^{-/-}$ recipient compared to a WT recipient. Data from the same mice as Figure 4.

D) Cellularity of peripheral blood CD3+ T cells in WT or IL7Rα$^{-/-}$ mice (solid bars) are compared to WT and IL7Rα$^{-/-}$ mice reconstituted with WT HSCs (pattern bars).

Differences in A-C’) were analyzed with two-tailed Student t-test relative to WT mice, and differences in D) were analyzed across all four conditions by one-way ANOVA ($p=0.0037$) and Dunnett multi-parameter test. *, $P<0.05$; **, $P< 0.005$; ***, $P< 0.0005$; ****, $P< 0.00005$.

Development • Supplementary information

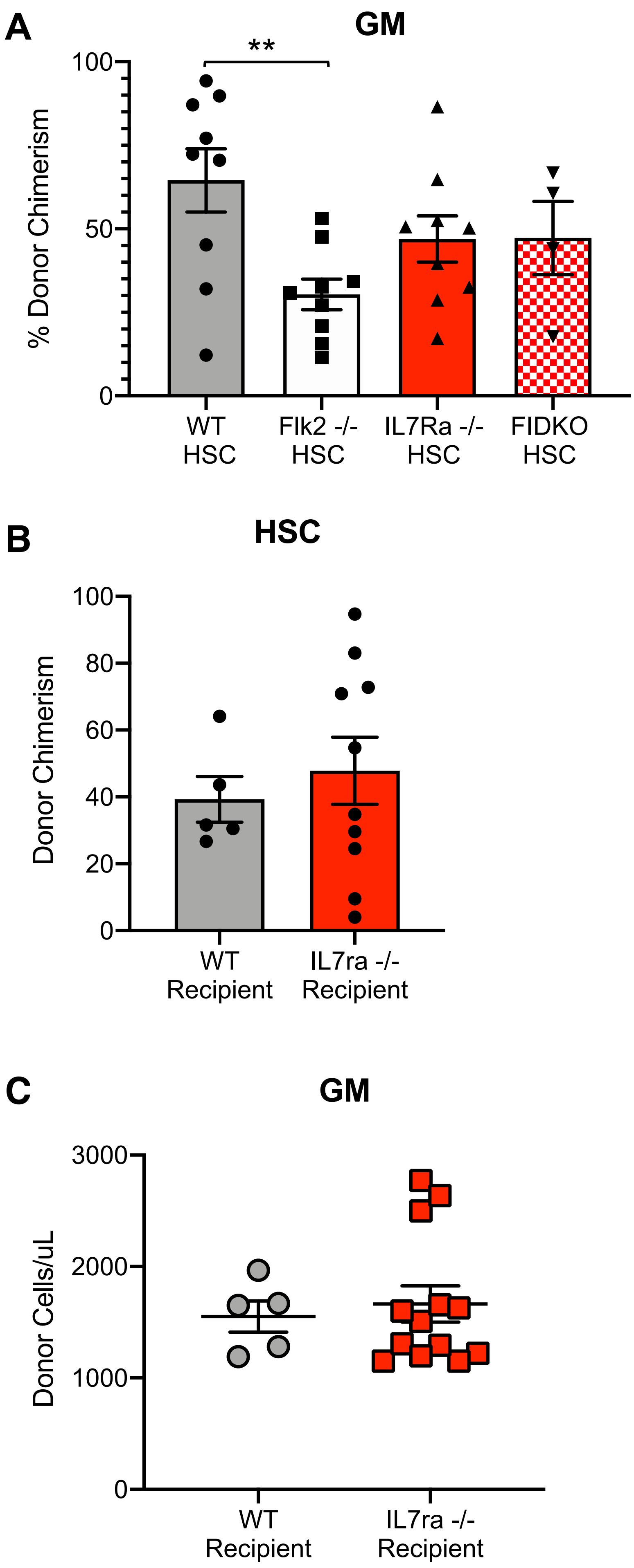

Development • Supplementary information

Fig. S3. HSC engraftment was robust, regardless of donor HSC or recipient genotype.
A) 500 WT, Flk2$^{-/-}$, IL7Rα$^{-/-}$ or FIDKO HSCs were transplanted into sublethally irradiated fluorescent WT recipients (mTmG or UBC-GFP) and donor chimerism of peripheral blood Granulocyte/Macrophages (GMs) cells was quantified 16 weeks post transplantation. Reconstitution of GMs was not significantly impaired upon transplantation of Flk2$^{-/-}$(white bars), IL7Rα$^{-/-}$ (red bars), or FIDKO HSCs (red/white bars) compared to WT HSCs (grey bars). Data from the same mice as in Figure 3.
B,C). 500 fluorescent WT HSCs (UBC-GFP), were transplanted into sublethally irradiated non-fluorescent WT or IL7Rα-/- recipients. Donor contribution to peripheral blood GMs and bone marrow HSCs was quantified 16 weeks post-transplantation. Data from the same mice as in Figure 4. **B)** Donor chimerism of bone marrow HSCs was similar in IL7Rα$^{-/-}$ compared to WT recipients. **C)** Donor-derived GM cells/uL of blood was similar in IL7Rα$^{-/-}$ compared to WT recipients. Statistics by two-tailed Student t-test. **, P< 0.005.

Development • Supplementary information

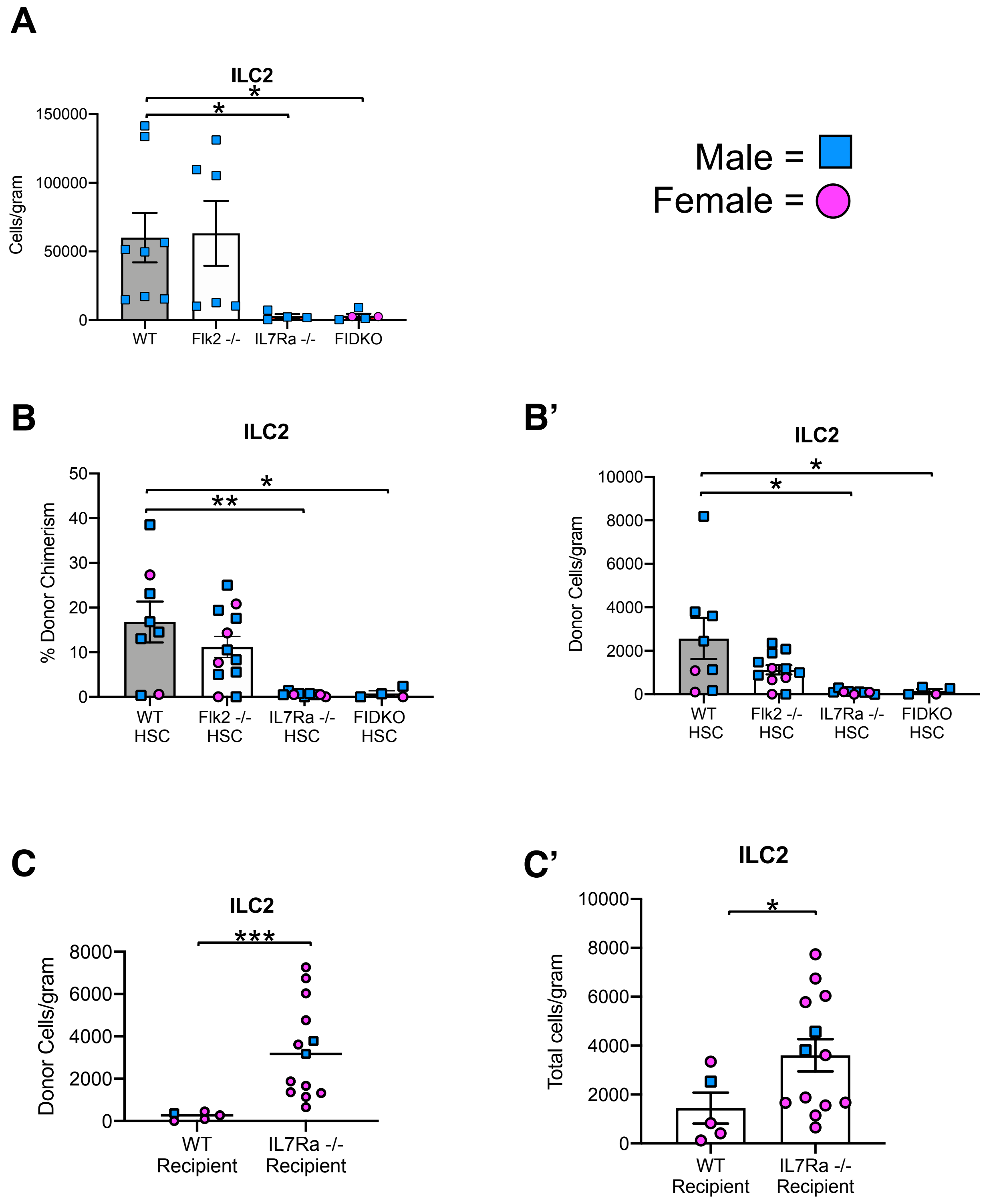

Fig. S4. ILC2s in the lung were similarly affected in male and female mice.
Data from figures 2, 3 and 4, with sex designation of each datapoint. Blue squares denote male mice and pink circles denote female mice.

A) Quantification of ILC2s in WT, Flk2$^{-/-}$, IL7Rα$^{-/-}$, and FIDKO mice. Data from Figure 2.

B) Donor Chimerism and

B') quantification of donor ILC2s in from WT HSC, Flk2$^{-/-}$ HSC, IL7Rα$^{-/-}$ HSC, and FIDKO HSC mice. Data from Figure 3.

C) Quantification of donor-derived ILC2s and

C') quantification of total (host+donor) ILC2 numbers in WT and IL7Rα$^{-/-}$ recipients. Data from Figure 4.

Development • Supplementary information

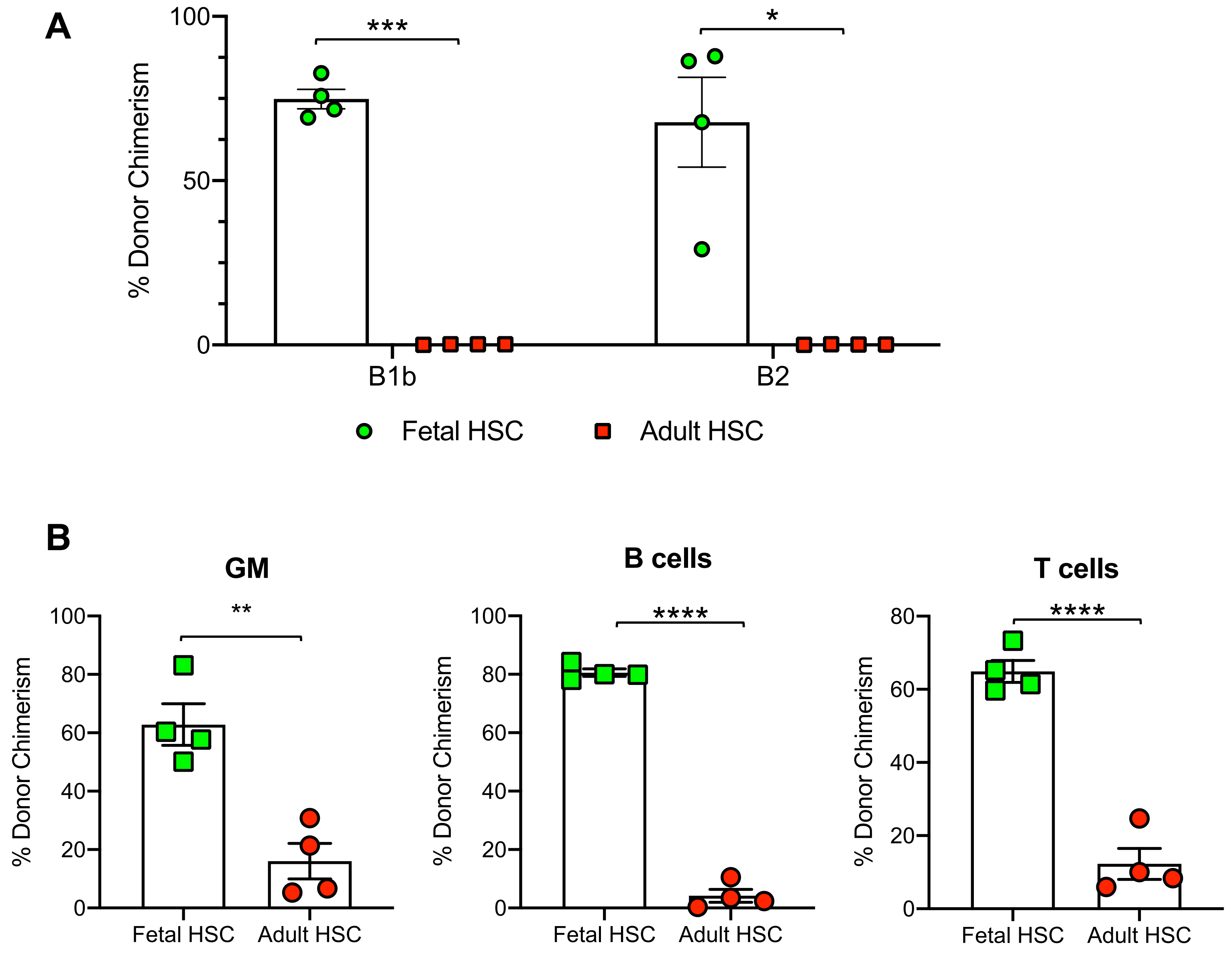

Fig. S5. Fetal HSCs had greater mature cell reconstitution capacity compared to adult HSCs. 250 fetal HSCs and 250 adult HSCs from different fluorescent mice (KuO and UBC- GFP, respectively) were co-transplanted into sublethally irradiated WT recipients and donor contribution to mature cells was quantified >16 weeks post transplantation.

A) Donor chimerism of peritoneal B1b and B2 was significantly greater by fetal HSCs compared to adult HSCs.

B) Donor chimerism of peripheral blood GMs, B cells and T cells was significantly greater by fetal HSCs compared to adult HSCs. Data from the same mice as in Figure 6. Differences were analyzed with two-tailed Student t test *, P<0.05; **, P< 0.005; ***, P<0.0005.

Development • Supplementary information

Table S1. Antibodies used in experiments

Antibody	Source	Identifier	Dilution
B220 - APC Cy7	Biolegend	103224	1:400
CD3- APC	Biolegend	100236	1:200
CD4 - BV605	Biolegend	100451	1:400
CD5 - APC	Biolegend	100625	1:200
CD11b - PB	Biolegend	101223	1:400
CD11b- PECy7	Biolegend	101216	1:400
CD21 - PE Cy7	Ebiosciences	25-0211-82	1:200
CD23 - PB	Biolegend	101616	1:200
CD25 - APC Cy7	Biolegend	102026	1:200
CD45.2 - PB	Biolegend	109820	1:400
CD61-Alexa 647	Biolegend	104314	1:400
CD150 - BV786	Biolegend	115937	1:400
cKit - APC Cy7	Biolegend	105826	1:800
Flk2 - APC	Biolegend	135310	1:100
FoxP3 - PB	Biolegend	126409	1:100
Gr1-Pacific Blue	Biolegend	108430	1:400
IgM - BV605	Biolegend	406523	1:400
KLRG - BV605	Biolegend	138419	1:100
Sca1 - PB	Biolegend	122520	1:400
TCRB - Pe Cy7	Biolegend	109222	1:200
Ter119-PECy5	Biolegend	116210	1:800

Antibody	Source	Identifier	Dilution
CD3 - A700	Biolegend	100216	1:100
CD4 - A700	Biolegend	100429	1:400
CD5 - A700	R&D Systems	FAB115N-025	1:400
CD8 - A700	Biolegend	300919	1:200
Ter119 - A700	Biolegend	116220	1:400
B220 - A700	Biolegend	103231	1:400
Gr1 - A700	Biolegend	108421	1:400
CD11b - A700	Biolegend	101222	1:800
CD3 - biotin	Biolegend	100243	1:200
CD4 - biotin	Biolegend	100403	1:200
CD5 - biotin	Biolegend	100603	1:400
CD8 - biotin	Biolegend	100703	1:200
Ter119 - biotin	Biolegend	116203	1:400
Nk1.1 - biotin	Biolegend	108703	1:200
CD19 - biotin	Biolegend	115503	1:400
F4/80 - biotin	Biolegend	123105	1:400
FcεRIα - biotin	Biolegend	101303	1:200
STA - BV786	Biolegend	405249	1:400

Development • Supplementary information
