# Supplementary material for: IL7Rα, but not Flk2, is required for hematopoietic stem cell reconstitution of tissue-resident lymphoid cells
Source: Development. 2022 Jan 24;149(8):dev200139. doi: 10.1242/dev.200139 (PMC8917444; doi:10.1242/dev.200139)
Supplement: Reviewer comments [file develop-149-200139_review_history.pdf]

IL7Rα, but not Flk2, is required for hematopoietic stem cell reconstitution of tissue-resident lymphoid cells

Atesh K Worthington, Taylor Cool, Donna M Poscablo, Adeel Hussaini, Anna E Beaudin and E Camilla Forsberg

DOI: 10.1242/dev.200139

Editor: Florent Ginhoux

Review timeline

Original submission: 26 August 2021
Editorial decision: 25 October 2021
First revision received: 7 November 2021
Accepted: 14 December 2021

Original submission

First decision letter

MS ID#: DEVELOP/2021/200139

MS TITLE: IL7Rα is required for hematopoietic stem cell reconstitution of tissue-resident lymphoid cells

AUTHORS: Atesh Worthington, Taylor Cool, Donna Poscablo, Adeel Hussaini, Anna Beaudin, and Camilla Forsberg

I have now received all the referees' reports on the above manuscript, and have reached a decision. The referees' comments are appended below, or you can access them online: please go to BenchPress and click on the 'Manuscripts with Decisions' queue in the Author Area.

As you will see, the referees express considerable interest in your work, but have some significant criticisms and recommend a substantial revision of your manuscript before we can consider publication. If you are able to revise the manuscript along the lines suggested, which may involve further experiments, I will be happy receive a revised version of the manuscript. Your revised paper will be re-reviewed by one or more of the original referees, and acceptance of your manuscript will depend on your addressing satisfactorily the reviewers' major concerns. Please also note that Development will normally permit only one round of major revision.

We are aware that you may be experiencing disruption to the normal running of your lab that make experimental revisions challenging. If it would be helpful, we encourage you to contact us to discuss your revision in greater detail. Please send us a point-by-point response indicating where you are able to address concerns raised (either experimentally or by changes to the text) and where you will not be able to do so within the normal timeframe of a revision. We will then provide further guidance. Please also note that we are happy to extend revision timeframes as necessary.

Please attend to all of the reviewers' comments and ensure that you clearly highlight all changes made in the revised manuscript. Please avoid using 'Tracked changes' in Word files as these are lost in PDF conversion. I should be grateful if you would also provide a point-by-point response detailing how you have dealt with the points raised by the reviewers in the 'Response to Reviewers' box. If you do not agree with any of their criticisms or suggestions please explain clearly why this is so.

© 2022. Published by The Company of Biologists under the terms of the Creative Commons Attribution License (https://creativecommons.org/licenses/by/4.0/).

Reviewer 1

Advance summary and potential significance to field

In Worthington et al., the authors examined how the loss of Flk2 and IL7 signaling affects the development of tissue lymphoid cells (TLCs), such as B1a, MZ B, and lung ILC2s and Tregs. Using a labeling system they have previously established, they show that nearly all TLCs derive from cells that expressed Flk2 and IL7. Furthermore, when examining Flk2 and IL7Ra KO mice, they find a dramatic loss of TLCs (except for Tregs), but only a mild loss of TLCs in the absence of Flk2. When transplanting KO HSCs into Wt recipients, they show a similar phenotype as the KO mice, indicating these effects are likely mostly cell-intrinsic. However, when transplanting Wt HSCs into IL7Ra KO recipients, they show TLC reconstitution even beyond what they observed in Wt recipients suggesting an overcompensation in the IL7Ra KO mice (likely due to excess IL7, as they later suggest). However, in most cases the reconstitution of TLCs falls far short of the steady-state levels of these cell types, indicating that adult HSCs are not able to fully reconstitute TLCs, a notion consistent with an embryonic contribution of many TLCs.

The strengths of this manuscript lie in the simple, straightforward, yet elegant experiments, and clear presentation of the results makes it easy to analyze their data. Their strategy is for absolute number quantification is clever (adding a known quantity of counting beads). The succinct supplementary data section is also greatly appreciated. My main concern is how much this adds to our current understanding, as the experiments are largely descriptive and many of their results could be explained by mechanisms that are not directly related to TLCs. This is not the fault of the research team, but a cohesive model that ties in their new findings to their prior results with their proposed mechanisms for the roles of Flk2 and IL7 in lymphocyte development would be helpful. Overall, I find this a solid study that is easy to read and understand, but would be improved with more attention to mechanism.

Comments for the author

Major concerns:
As mentioned above, a final figure with a model would be helpful, linking this current work to their prior work. If not a figure, then just a more detailed summary in the discussion section.
IL7 signaling is critical for early lymphocyte development at the CLP and early B cell stages. It is unclear if TLCs require IL7 beyond these stages or are the deficiencies the authors observe due to lack of survival/expansion at the CLP stage? Much of the TLC data is similar to that in B2 B cells. Do the authors feel their data indicate downstream roles for IL7 in TLC-specific differentiation or homeostasis? These questions might be answered by conditional KOs at later stages (e.g. CD19-Cre), but that would be more appropriate for a future study.

Minor concerns:
The transplantation system is a bit non-standard (sublethal 750 rads), and it would be helpful to explain the system to readers to better understand the role the host cells play in terms of competition with the donor cells. At 750 Rads, I assume all host lymphocytes are ablated, and so any host lymphocytes would be newly derived from host HSCs that survive the irradiation. Is this correct? The authors do not include any reconstitution data (e.g. BM KLS, or blood granulocytes), so it's difficult to know how strong the HSC reconstitution was.
Much of the uncertainty in lineage reconstitution in those systems could be solved (potentially) by a competitive transplantation of Wt and IL7Ra KO HSCs. Would a competitive transplantation system with an equal number of Wt and IL7Ra KO HSCs be more informative?
How does their Treg data compare to conventional CD4+ T cells? Were there any differences that might indicate a unique requirement of Flk2 or IL7 signaling in Tregs?
It's not clear whether the Wt/IL7Ra KO recipient data in Figure 5 is the same data as in Figure 4. The differences in reconstitution highlighted in Figure 4 does not seem present in the recipient data in Figure 5. Please clarify.
Fix error bars on Figure 5A (one sided) so they look the same as the other error bars in that Figure (two sided).
The authors could amend their title to "IL7Ra, but not Flk2, is required for,…" if they were so inclined.
Typo on line 106

© 2022. Published by The Company of Biologists under the terms of the Creative Commons Attribution License (https://creativecommons.org/licenses/by/4.0/).

Reviewer 2

Advance summary and potential significance to field

The study by Worthington et al. advances basic understanding of the developmental requirements of tissue-resident lymphocytes, especially for regulatory T cells.
It shows a critical intrinsic role for IL7 signaling and a partial need for Flt3 signaling, and how adult-derived HSCs cannot fully replenish the tissue-resident lymphocyte compartment after transplantation in adult recipients, which may have clinical relevance.

Comments for the author

Essential revisions:
1. Can the authors elaborate on the absolute cell counting method? How is this different or improved from calculating absolute cell numbers from hematocytometer cell counting for total cell numbers and % of populations quantified by flow cytometry?
2. The authors describe adult WT transplant studies. Have parallel studies been performed with fetal HSCs or neonatal HSCs? If so, what is the capacity of the fetal or neonatal progenitors to reconstitute the TLCs?
3. Please describe how many males and females were used for each group, and if there are any differences in the result when disaggregated by sex.
4. The data are mostly displayed as bar graphs showing the mean and SEM. This limits the the reader from evaluating the variance in the data points; this is reflected in the standard deviation (SD). The SEM is a indication of how far the calculated mean is from the ?true mean?, and is always smaller than the SD.
Therefore reporting the SEM only can be misleading when evaluating data. The authors should edit the graphs to be similar to the graphs shown in Figure 4 where all points are shown. This would make it clear to the reader the total number of points in each group, as well as an idea of the spread of the data points. In addition, the authors should also explain how the minimum number of animals in each group was determined, using a power analysis.
5. T-tests are best utilized to compare two groups, whereas ANOVA is used to compare across 3 or more groups. The authors used T-tests and ANOVAs are used to compare differences across groups, but these tests seem to be used inconsistently for each Figure. For example, t-tests are used in Figures 1-4 but only ANOVA is used in Figure 5, but only t-tests are used in Figure S1E, and S1E? (where it seems ANOVA would be appropriate, as these data are related to the data in Figure 5). The authors should explain why they chose the statistical tests they used.

Problems/Limitations
1. The study findings, although novel, are incremental. Even the authors highlight that their results confirm previous findings from other groups.
Although this is encouraging from the reproducibility aspect, the report as a whole makes an incremental advance in the field. Some follow up on molecular mechanisms on the inability of Flk2 to compensate for IL7Ra in the TLCs would strengthen the manuscript. Could the authors follow up on even just one TLCs subset (e.g. Tregs, ILC2s, to investigate how Flk2 is promoting greater numbers in the IL7Ra-/-?)

2. The cell extrinsic role of IL7Ra signaling to inhibit the numbers of ILCs and Tregs in the WT->IL7Ra chimeras is interesting, but no mechanisms are defined to explain the changes. The authors allude to other cytokines that may be changed (Cool et al. 2020). Could the author expand on this by defining these ?other cytokine changes? in the current manuscript in relation to ILC2s and Tregs?

Minor comments:
1. Line 106: The word ?a? should be deleted.
2. Line 403: there is a typo: C57BL/6 is the correct nomenclature.

© 2022. Published by The Company of Biologists under the terms of the Creative Commons Attribution License (https://creativecommons.org/licenses/by/4.0/).

First revision

Author response to reviewers' comments

We thank the Reviewers for their positive comments and constructive feedback. Below, we provide a point-by-point response to how we have improved the manuscript by adding new results (**new Figures 6 and 7, and new Supplemental Figures S2-S5**) and clarifying and expanding the text. We hope that these additions further increase the Reviewers' enthusiasm for publishing these findings in *Development*.

Reviewer 1 Advance Summary and Potential Significance to Field: In Worthington et al., the authors examined how the loss of Flk2 and IL7 signaling affects the development of tissue lymphoid cells (TLCs), such as B1a, MZ B, and lung ILC2s and Tregs. Using a labeling system they have previously established, they show that nearly all TLCs derive from cells that expressed Flk2 and IL7. Furthermore, when examining Flk2 and IL7Ra KO mice, they find a dramatic loss of TLCs (except for Tregs), but only a mild loss of TLCs in the absence of Flk2. When transplanting KO HSCs into Wt recipients, they show a similar phenotype as the KO mice, indicating these effects are likely mostly cell-intrinsic. However, when transplanting Wt HSCs into IL7Ra KO recipients, they show TLC reconstitution even beyond what they observed in Wt recipients, suggesting an overcompensation in the IL7Ra KO mice (likely due to excess IL7, as they later suggest). However, in most cases the reconstitution of TLCs falls far short of the steady-state levels of these cell types, indicating that adult HSCs are not able to fully reconstitute TLCs, a notion consistent with an embryonic contribution of many TLCs.

The strengths of this manuscript lie in the simple, straightforward, yet elegant experiments, and clear presentation of the results makes it easy to analyze their data. Their strategy is for absolute number quantification is clever (adding a known quantity of counting beads). The succinct supplementary data section is also greatly appreciated. My main concern is how much this adds to our current understanding, as the experiments are largely descriptive and many of their results could be explained by mechanisms that are not directly related to TLCs. This is not the fault of the research team, but a cohesive model that ties in their new findings to their prior results with their proposed mechanisms for the roles of Flk2 and IL7 in lymphocyte development would be helpful. Overall, I find this a solid study that is easy to read and understand, but would be improved with more attention to mechanism.

Reviewer 1 Comments for the Author:
Major concerns: As mentioned above, a final figure with a model would be helpful, linking this current work to their prior work. If not a figure, then just a more detailed summary in the discussion section.

Response: We thank the Reviewer for the positive comments on our manuscript and for the suggestion to add a model figure. We agree that this would further enhance the impact of the study, and have now added such a figure (**new Figure 7**), along with a discussion that ties the new findings to previous work (pages 12-15).

IL7 signaling is critical for early lymphocyte development at the CLP and early B cell stages. It is unclear if TLCs require IL7 beyond these stages, or are the deficiencies the authors observe due to lack of survival/expansion at the CLP stage? Much of the TLC data is similar to that in B2 B cells. Do the authors feel their data indicate downstream roles for IL7 in TLC-specific differentiation or homeostasis? These questions might be answered by conditional KOs at later stages (e.g. CD19-Cre), but that would be more appropriate for a future study.

Response: We agree with the Reviewer that these are important questions, but also with the sentiment that additional lineage tracing models are more appropriate for future studies. We have added a discussion of cytokines (pages 14-15), the Reviewer's idea of lineage-specific deletion to the discussion of future directions (page 15), and also a model figure (**new Figure 7**) to illustrate the role of Flk2 and IL7R in partially overlapping progenitor cells.

© 2022. Published by The Company of Biologists under the terms of the Creative Commons Attribution License (https://creativecommons.org/licenses/by/4.0/).

Minor concerns:
The transplantation system is a bit non-standard (sublethal 750 rads), and it would be helpful to explain the system to readers to better understand the role the host cells play in terms of competition with the donor cells. At 750 Rads, I assume all host lymphocytes are ablated, and so any host lymphocytes would be newly derived from host HSCs that survive the irradiation. Is this correct? The authors do not include any reconstitution data (e.g. BM KLS, or blood granulocytes), so it's difficult to know how strong the HSC reconstitution was.

Response: We appreciate the Reviewer's request for additional information and have added the relevant information to the revised manuscript (page 8), with reference to the extent of host cell depletion with sublethal and lethal irradiation (Boyer et al, SCR 2019). In short, not even lethal irradiation depletes all host lymphocytes, nor all HSCs. This is reflected in reconstitution experiments, where a percentage of, rather than all, cells are donor-derived.

To address the level of HSC reconstitution, we have added new panels of peripheral blood GM chimerism and bone marrow HSC chimerism (**new Figure S3**, with text on pages 8-9 and 15).

Much of the uncertainty in lineage reconstitution in those systems could be solved (potentially) by a competitive transplantation of Wt and IL7Ra KO HSCs. Would a competitive transplantation system with an equal number of Wt and IL7Ra KO HSCs be more informative?

Response: We appreciate this suggestion, but in our experience competitive transplantations are valuable primarily in cases where differences in reconstitution are very subtle. In the case of IL7Ra KO HSCs, the reconstitution of lymphocytes is clearly evident. It is also highly specific for lymphocytes, but not myeloid cells, as shown in the new results we added (**new Figure S3**). Thus, we can conclude that lymphoid reconstitution is severely and selectively impaired without performing competitive transplantations.

How does their Treg data compare to conventional CD4+ T cells? Were there any differences that might indicate a unique requirement of Flk2 or IL7 signaling in Tregs?

Response: We thank the Reviewer for these questions as we agree that they are important comparisons to make. We did not have data on CD4+ cells, but have added data of conventional CD3+ T cells from the peripheral blood in **new Figure S2**. Briefly, Tregs and conventional T cells are similarly affected by Flk2, IL7R, and combined deletion at steady-state (compare Figure 2F to S2A) and upon transplantation (Figure 3F versus S2B). Upon transplantation of WT HSCs into $Il7ra^{-/-}$ hosts, Tregs, but not circulating T cells, displayed increased engraftment (Figure 4E and E' versus S2C and S2C') and we only observed full rescue to steady-state numbers of conventional T cells, and not Tregs, upon transplantation of WT HSCs in WT and $IL7ra^{-/-}$ recipients (Figure 5E versus S2D). An overview of these data is now also summarized in the requested model figure (**new Figure 7**).

It's not clear whether the Wt/IL7Ra KO recipient data in Figure 5 is the same data as in Figure 4. The differences in reconstitution highlighted in Figure 4 does not seem present in the recipient data in Figure 5. Please clarify.

Response: We apologize for the confusion. The data in Figure 4B', C', D' and E' are present in the recipient data in Figure 5B, C, D and E, displayed as the bars with grey and red stripes. Figure 5 also contains additional data. We performed t-tests for Figure 4 as there are only two comparisons, but ANOVA for Figure 5 because there are multiple comparisons. By t-test, the significant differences in total ILC2 and Tregs (donor+host) that were highlighted in Figure 4 are now denoted by the grey asterisk in Figure 5 and have been clarified in the figure legend.

Fix error bars on Figure 5A (one sided) so they look the same as the other error bars in that Figure (two sided).

Response: Thank you for this suggestion. We have replotted this panel.

© 2022. Published by The Company of Biologists under the terms of the Creative Commons Attribution License (https://creativecommons.org/licenses/by/4.0/).

The authors could amend their title to “IL7Ra, but not Flk2, is required for,…” if they were so inclined.

Response: This is a great suggestion and we have added this mention of Flk2 to the title to better emphasize to readers that Flk2 was studied in addition to IL7Ra.

Typo on line 106

Response: Thank you; we have corrected this (page 4).

Reviewer 2 Advance Summary and Potential Significance to Field:
The study by Worthington et al. advances basic understanding of the developmental requirements of tissue-resident lymphocytes, especially for regulatory T cells. It shows a critical intrinsic role for IL7 signaling and a partial need for Flt3 signaling, and how adult-derived HSCs cannot fully replenish the tissue-resident lymphocyte compartment after transplantation in adult recipients, which may have clinical relevance.

Reviewer 2 Comments for the Author: Essential revisions:

1. Can the authors elaborate on the absolute cell counting method? How is this different or improved from calculating absolute cell numbers from hematocytometer cell counting for total cell numbers and % of populations quantified by flow cytometry?

Response: We appreciate the request for additional information, and have expanded our statement on page 8 to further clarify. In short, host cell depletion and variable recovery of different cell types in different recipient mice, influences chimerism data, but not total donor cell number data. This variability can mask statistically significant differences in host cell reconstitution, as exemplified in Figure 3 for the $Flk2^{-/-}$ results. These concepts were initially put forth in a research report from our lab (Boyer et al 2019), included in a subsequent methods paper (Rajendiran et al 2020) and utilized in two recent research papers where the approach was favorably reviewed (Cool et al 2020; Poscablo et al 2021). These are all referenced in the text and methods sections of the revised manuscript.

The rationale for using beads and flow cytometry as opposed to hemocytometer is simply speed and simplicity: we perform the cell counting at the same time as we perform the cell type- and donor/host- specific analysis. This simple method avoids additional sample preparation and therefore risk of introduction of additional variably.

2. The authors describe adult WT transplant studies. Have parallel studies been performed with fetal HSCs or neonatal HSCs? If so, what is the capacity of the fetal or neonatal progenitors to reconstitute the TLCs?

Response: We appreciate the Reviewer’s questions regarding fetal progenitor TLC reconstitution and believe these are important and controversial questions based on the proposed fetal origin of some of these TLCs and lack of clear consensus for all of them. Therefore, we have done our best to address this question by performing competitive transplants of fetal and adult HSCs and examining TLC reconstitution in the same recipients to be able to directly compare the TLC reconstitution capacity of fetal and adult HSCs. We are very excited to share these new data with the addition of two figures, **new Figures 6 and S5**. Briefly, we find that in the same recipient, fetal HSCs outperformed adult HSCs in TLC reconstitution. Mechanistically, this is not due to differences in HSC engraftment (**new Figure 6C**), but likely due to lymphoid bias of fetal HSCs relative to adult HSCs. This is apparent in significant differences in CLP reconstitution (**new Figure 6C**), but lack of differences in myeloid reconstitution (CMPs, **new Figure 6C,** text on pages 11 and 13-15). We believe these new data adds to the impact of our current findings and will be suited for follow up investigation in future studies.

© 2022. Published by The Company of Biologists under the terms of the Creative Commons Attribution License (https://creativecommons.org/licenses/by/4.0/).

3. Please describe how many males and females were used for each group, and if there are any differences in the result when disaggregated by sex.

Response: Thank you for this suggestion. We have included the numbers of males and females in each of the legends of relevant figures. We are aware of one report of sex differences in ILC2 cells. Therefore, we have now also plotted the ILC2 data with sex indicated and included this as new **Figure S4,** with text on page 9. These analyses did not reveal any sex-dependent trends or differences.

4. The data are mostly displayed as bar graphs showing the mean and SEM. This limits the reader from evaluating the variance in the data points; this is reflected in the standard deviation (SD). The SEM is a indication of how far the calculated mean is from the ?true mean?, and is always smaller than the SD. Therefore reporting the SEM only can be misleading when evaluating data. The authors should edit the graphs to be similar to the graphs shown in Figure 4, where all points are shown. This would make it clear to the reader the total number of points in each group, as well as an idea of the spread of the data points. In addition, the authors should also explain how the minimum number of animals in each group was determined, using a power analysis.

Response: We thank the Reviewer for this suggestion and agree that the Figure 4 data display was the clearest. We have now replotted the data for the other figures, as suggested by the Reviewer. We also included the requested information on power analysis in the methods section on page 20.

5. T-tests are best utilized to compare two groups, whereas ANOVA is used to compare across 3 or more groups. The authors used T-tests and ANOVAs are used to compare differences across groups, but these tests seem to be used inconsistently for each Figure. For example, t-tests are used in Figures 1-4, but only ANOVA is used in Figure 5, but only t-tests are used in Figure S1E, and S1E'? (where it seems ANOVA would be appropriate, as these data are related to the data in Figure 5). The authors should explain why they chose the statistical tests they used.

Response: We appreciate the Reviewer's comment and agree that ANOVA should be used for Figures S1E and S1E', as we had done for Figure 5. We have updated Figures S1E and S1E' and the figure legend to reflect these changes (page 34). We used T-tests in Figures 2-4, 6, S2A-C', S3-S5 as we only compared our experimental groups to our WT controls, even though all experimental groups are graphed together for each cell type. We intentionally graphed all experiment groups together to avoid having figures with 27+ panels. We apologize for the confusion, and have further explained this reasoning in the methods on page 20.

Problems/Limitations

1. The study findings, although novel, are incremental. Even the authors highlight that their results confirm previous findings from other groups. Although this is encouraging from the reproducibility aspect, the report as a whole makes an incremental advance in the field. Some follow up on molecular mechanisms on the inability of Flk2 to compensate for IL7Ra in the TLCs would strengthen the manuscript. Could the authors follow up on even just one TLCs subset (e.g. Tregs, ILC2s, to investigate how Flk2 is promoting greater numbers in the IL7Ra-/-?)

Response: We thank the Reviewer for acknowledging that our findings are novel, and we agree that some of them are not unexpected, given previous complementary studies. We have not been able to pinpoint a molecular mechanism that explain how Flk2 fails to compensate for IL7Rα. In the context of transplantation into IL7Rα$^{-/-}$ mice, we believe that host upregulation of IL7, rather than Flk2 signaling, increase TLC reconstitution over that in WT mice (also see below and edits on page 14 of the manuscript). In the revised manuscript, we have added data that helps understand the cellular mechanisms and lineage selectivity of Flk2 and IL7Rα (for example, addition of HSC engraftment and GM reconstitution data in **new Figure S3**). We hope that these data, along with the additional new results presented in **new Figures 6-7** and **S2-S5** and the expansion and clarification of the text, will further increase the Reviewers' enthusiasm for recommending the study for publication.

2. The cell extrinsic role of IL7Ra signaling to inhibit the numbers of ILCs and Tregs in the WT->IL7Ra chimeras is interesting, but no mechanisms are defined to explain the changes. The authors

© 2022. Published by The Company of Biologists under the terms of the Creative Commons Attribution License (https://creativecommons.org/licenses/by/4.0/).

allude to other cytokines that may be changed (Cool et al. 2020). Could the author expand on this by defining these ?other cytokine changes? in the current manuscript in relation to ILC2s and Tregs?

Response: We appreciate this suggestion and have explored additional candidate cytokines. Unfortunately, we have not uncovered robust upregulation of lymphoid-promoting factors other than IL7 (as already reported by us and others) and the other cytokines referred to in Cool et al, 2020, were downregulated. This leaves IL7 as the current sole candidate for the increased reconstitution in IL7Rα$^{-/-}$ mice over WT recipients. We have edited the text (page 14) to clarify this to the readers. The role of IL7 signaling in tissue immune cells will be pursued in the future by one of the coauthors (Dr. Beaudin), who has secured an R01 for this project in her own laboratory.

Minor comments:

1. Line 106: The word ?a? should be deleted.

Response: Thank you; we have corrected this (Line 106 on page 4).

2. Line 403: there is a typo: C57BL/6 is the correct nomenclature.

Response: Thank you; we have corrected this (now Line 457 on page 17).

Second decision letter

MS ID#: DEVELOP/2021/200139

MS TITLE: IL7Rα, but not Flk2/Flt3, is required for hematopoietic stem cell reconstitution of tissue-resident lymphoid cells

AUTHORS: Atesh Worthington, Taylor Cool, Donna Poscablo, Adeel Hussaini, Anna Beaudin, and Camilla Forsberg
ARTICLE TYPE: Research Article

I am happy to tell you that your manuscript has been accepted for publication in Development, pending our standard ethics checks.

Reviewer 1

Advance summary and potential significance to field

The advance of this paper is increasing our understanding of the developmental pathways by which tissue resident immune populations arise. While traditional lymphocytes arise from a common lymphoid progenitor and relies on Flk2 and IL7 signaling, tissue-resident lymphocyte cells (TLCs) have a more complicated ontogeny. Using an elegant series of lineage-tracing and depletion experiments, the authors work adds to the body of knowledge on TLC development, and show the requirement of IL7, but not Flk2 signaling.

Comments for the author

The revised manuscript appears to address the bulk of the reviewers' concerns. I appreciate they modified their figures to show individual data points, and their statistical analyses to be more consistent and appropriate. The new figure 6 showing competitive repopulation between fetal and adult HSCs is interesting and informative, and in my opinion strengthens the manuscript (I'm assuming they used a paired T-test for the competitive transplants). I appreciate the added model Figure 7, although it's more a summary of their results than a model of what they think is going on. My key concerns have been addressed by their revisions.

© 2022. Published by The Company of Biologists under the terms of the Creative Commons Attribution License (https://creativecommons.org/licenses/by/4.0/).

Reviewer 2

Advance summary and potential significance to field

The study by Worthington et al. advances basic understanding of the developmental requirements of tissue-resident lymphocytes, especially for regulatory T cells.
It shows a critical intrinsic role for IL7 signaling and a partial need for Flt3 signaling, and how adult-derived HSCs cannot fully replenish the tissue-resident lymphocyte compartment after transplantation in adult recipients, which may have clinical relevance.

Comments for the author

The revisions to the manuscript are excellent. I recommend publication.

© 2022. Published by The Company of Biologists under the terms of the Creative Commons Attribution License (https://creativecommons.org/licenses/by/4.0/).
